# Investigating Cell Wall Diffusion in Wood Modified with Phenol Urea Formaldehyde Resin in Different Length Scales

**DOI:** 10.1021/acs.biomac.4c01168

**Published:** 2025-01-14

**Authors:** Carlo Kupfernagel, Mohammed Rahman, Rosalie Cresswell, Morwenna J. Spear, Andrew Pitman, Steven P. Brown, Graham A. Ormondroyd

**Affiliations:** † Institut für Holztechnologie Dresden gGmbH, 01217 Dresden, Germany; ‡ Department of Physics, University of Warwick, Coventry CV4 7AL, U.K.; § BioComposites Centre, Bangor University, Bangor LL57 2UW, U.K.; ∥ BM Trada, Buckinghamshire HP14 4ND, U.K.

## Abstract

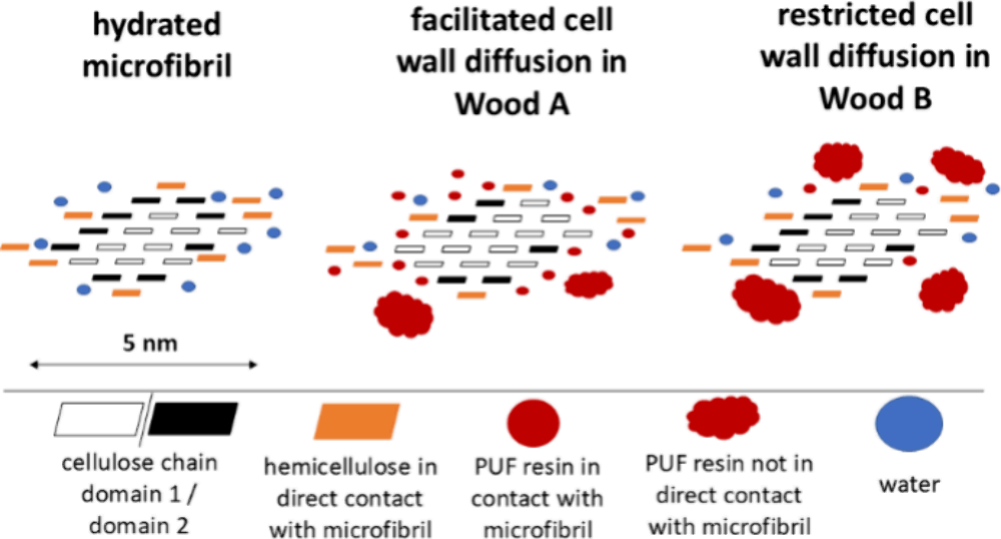

Wood modification using low molecular weight thermosetting resins improves the biological durability and dimensional stability of wood while avoiding increasingly regulated biocides. During the modification process, resin monomers diffuse from the cell lumen to the cell wall, occupying micropore spaces before *in situ* curing at 150 °C. This study investigated the mechanism of cell wall diffusion at multiple scales, comparing two test groups where diffusion was either facilitated or restricted. Antiswelling efficiency tests demonstrated improved dimensional stability when diffusion was facilitated. Differential scanning calorimetry showed that bound water was excluded more effectively from the cell wall if cell wall diffusion was enabled. Solid-state NMR spectroscopy (^1^H MAS and ^13^C MAS) with relaxation time analysis indicated that resin migrated to distinct locations within the cell wall, influenced by diffusion and drying conditions. These findings highlight how optimizing cell wall diffusion can significantly improve the performance of wood modification processes using thermosetting resins.

## Introduction

Wood is one of the oldest building materials utilized by mankind, and its environmental benefits in comparison to other nonrenewable or fossil-based materials is widely recognized today as part of a sustainable future.^1^ However, the use of timber is limited in certain applications, such as those requiring a high biological durability or dimensional stability. Durability issues have long been addressed using conventional preservatives, such as copper-chrome-arsenate or creosote. These compounds increase the durability of wood using a fungicidal mode of action, which increasingly restricts their use within close human contact. Alternative wood protection methods with a nonfungicidal mode of action are frequently summarized as wood modification.^2−4^ Wood modification with low molecular weight thermosetting resin (e.g., phenol urea formaldehyde, furfuryl alcohol) is a frequently studied technique that is suitable for upscaling,^5−7^ and while certain studies have provided insight into the interactions between wood and resin molecules,^8^ a comprehensive understanding of these interactions at the nanoscale remains to be fully explored. The treatment with PUF resin typically involves an impregnation, drying, and heat curing step. After impregnation, when the resin is uncured, the material is pliable and can easily be compressed, e.g., when manufacturing densified wood products.^9,10^ After heat curing, the material becomes rigid and loses its ability to undergo plastic deformation.^11^ This behavior indicates changes in the molecular architecture of the wood.

During impregnation, the bulk volume of the wood structure (i.e., cell lumen) is soaked with an aqueous solution containing low molecular weight monomers. The diffusion of resin monomers to the micropores in the cell wall, however, is an additional process to the bulk impregnation processes that relies on fluid flow within the cell lumena. Any reagent that is intended to react with or within the cell wall must be able to access these micropore spaces. Micropores are elongated voids between micro- and macrofibrils that are said to have a diameter between 2–4 nm.^12^ The common model for the cell wall diffusion to micropores depends on (1) a concentration gradient between the cell wall and cell lumen and (2) the mobility of the resin monomer.^13−17^ This model assumes that resin is initially distributed equally between cell wall and lumen after impregnation, i.e., without a concentration gradient.^13−17^ As water evaporates faster from the lumen than from the cell wall, a concentration gradient is introduced as soon as the wood starts drying. Resin monomers then diffuse from the place of high concentration (lumens) to the place of low concentration (micropores in the cell wall). The mobility of the resin monomer may relate to its kinetic energy and to the physical state of the wood polymers, hence, whether they are dry and glassy or hydrated and rubbery.^18^ Both the concentration gradient and the mobility of the monomers are affected by the atmospheric conditions in the drying stage, where a high temperature and high relative humidity facilitate cell wall diffusion, and low temperature and low humidity restrict cell wall diffusion.^14−17,19^ Once the wood is sufficiently dried, heat curing is initiated by temperatures up to 150 °C. Resin monomers that diffused into the micropores polymerize *in situ* and lock the cell wall in a permanently swollen state, making them less accessible to water. This mode of action is called micropore blocking.^20^

Resin deposits in micropores are in close contact with the interfaces of micro- and macrofibrils. Interactions at this length scale can be studied by solid-state nuclear magnetic resonance (NMR) spectroscopy and specifically relaxation time analysis, where different relaxation times, such as *T*_1_ (^1^H), *T*_1ρ_ (^1^H), and *T*_1_ (^13^C), correspond to distinct time scales of motions, and therefore, to different ranges of distance.^21,22^ The method is suitable to study morphological phase differences in polymers in the cell wall. Laborie argued that the spin–lattice time in the rotating frame *T*_1ρ_ and spin–lattice time *T*_1_ correspond to a morphological phase distance of 2–30 nm and >30 nm, respectively.^8^ The morphological phase size in the cell wall is such that spin diffusion is ineffective under certain conditions (e.g., in a wet state), which results in distinct relaxation times for different cell wall polymers.^23,24^ Therefore, one can use the presence or absence of spin-diffusion to study the morphological phases on different length scales. Spin-coupled carbons show identical *T*_1ρ_ values even if they are in a different chemical environment. The signals of cellulose domains 1 and 2 are typically spin-coupled, showing similar *T*_1ρ_ relaxation times.^8,24^

Cellulose domain 1 is mainly thought to be glucan chains in the core of the microfibril, hence interior cellulose, and has historically been referred to as crystalline cellulose. Cellulose domain 2 is primarily located on the surface of the microfibril, hence surface cellulose, and in the past has been assumed to be amorphous cellulose. In 1D solid-state NMR, these two domains are distinguished by different chemical shifts of their C4 and C6 signals. Laborie showed that neither heat treatments, mild alkali treatments, nor modification with high molecular weight resin disturbed the spin coupling of the two cellulose domains.^8^ However, the treatment with a low molecular weight resin caused significantly different *T*_1ρ_ values for domains 1 and 2, suggesting that resin monomers diffused close to the microfibril, interacting with its surface. Similar studies have been published more recently,^25,26^ but many aspects of the nanoscale interaction between wood and resin remain elusive, such as the nanometer scale location of cured resin in the wood structure or the accessibility for water in cured wood.

In the current study, we manipulate the conditions under which resin impregnated wood is dried to either facilitate (Wood A) or restrict (Wood B) cell wall diffusion. The macroscopic effect of these differences in cell wall diffusion is investigated using an antiswelling efficiency (ASE) test. Differential scanning calorimetry (DSC) is used to estimate the amount of bound water before and after modification. Solid-state NMR and relaxation time analyses show how diffusion conditions affect the molecular motions of wood polymers.

## Experimental Section

### Material

The sapwood of tulipwood (*Liriodendron tulipifera*) was cut into specimens with the dimensions of 20 (radial) × 20 (tangential) × 5 (longitudinal) mm. After impregnation, the samples were weighed to determine the liquid resin uptake (LU). All specimens were cut from a single strip of wood to minimize the variability. For each of the three test groups (Wood A, Wood B, and Control), 20 specimens were randomly selected from within this strip. Commercial PUF-resin (Prefere 5K600M) was provided by Prefere GmbH, Germany. Prior to impregnation, the resin was diluted with deionized water to a solids content of 20% and pH 9.2. The molecular weight information as reported by Prefere is *M*_n_ = 406 g/mol and *M*_w_ = 484 g/mol. Potassium hydroxide (KOH) is present as an alkaline catalyst.

### Resin Treatment and ASE Tests

Batches of 20 specimens were oven-dried to determine their dry mass and dimensions in the unmodified state. Afterward, the specimens were stored at ambient conditions for 1 week before being vacuum impregnated with the PUF resin for 20 min. The subsequent drying procedure was performed in two different ways to either promote or restrict the cell wall diffusion. One set (Wood A) was placed in a sealed container over saturated sodium hydroxide (NaCl) solution and then placed in an oven set to 30 °C for the 7 day diffusion step (Figure S1). This corresponds to an RH of 75% during the diffusion step. The weight and dimensions of each sample were monitored daily. Two samples from the stock of Wood A were put aside after the diffusion step. These samples did not undergo heat curing and will later be termed “Wood A uncured ambient” in the NMR section. In the drying step, the sample holder with the remaining samples was transferred to another container over a saturated MgCl_2_ solution to maintain a relative humidity of 31%. For Wood A, this container was placed in an oven set to 50 °C for 1 day.

After impregnation, another set (Wood B) was placed in a sealed container over a saturated KOH solution and then placed in a fridge set to 4 °C for the 7 day diffusion step. This corresponds to a RH of 14% throughout the diffusion step, which decreased to 6% in the drying step due to the change in RH that is generated by the salt solution at different temperatures. Two samples from the stock of Wood B were put aside after the diffusion step and will later be termed “Wood B uncured ambient”. In the drying step, the whole container with the remaining samples was transferred to an oven set to 50 °C for 1 day.

The remaining specimens of Wood A and B were heat cured at 150 °C for 8 h. The moisture content (determined gravimetrically) before heat curing was 9.66% (±0.34) in Wood A and 5.67% (±0.10) in Wood B. Temperature measurements performed by thermocouples (Type K) in the core of one sample from each set are very similar, despite the different moisture contents prior to curing, indicating no major differences in the heat curing step (Figure S2). After heat curing, the samples were weighed and dimensions were noted to determine the bulking coefficient (BU) and the weight percentage gain (WPG). Some cured samples were put aside for later analysis. Two samples of Wood A and Wood B were put aside without undergoing ASE tests. These samples were oven-dried and will be referred to as “Wood A cured dry” and “Wood B cured dry” in the NMR section.

The other cured samples were subjected to three cycles of oven drying and water soaking to determine the swelling coefficients (*S*_1_, *S*_2_, and *S*_3_), the mass loss due to leaching (ML_1_), and the gravimetric water uptake (WU_1_). Details about the ASE method and calculations of LU, WPG, BU, *S*_1_, *S*_2_, *S*_3_, ML_1_, and WU_1_ are described elsewhere.^27^

### Scanning Electron Microscopy (SEM)

Wood specimens for SEM were prepared by smoothing the tangential–longitudinal faces and making several 10 μm cuts with a rotary microtome. Uncured samples were dry cut to avoid leaching of the uncured resin. Cured samples were softened by being soaked in water prior to microtome cutting. The small pieces were then dried and attached using carbon tape onto aluminum stubs without further pretreatment. All SEM images were taken with a scanning electron microscope (Hitachi, TM4000) using a beam acceleration voltage of 15 kV and a detector for backscattered electrons.

### Sample Preparation for Differential Scanning Calorimetry

Small solid wood sections of approximately 3 (radial) × 3 (tangential) × 3 (longitudinal) mm were cut in replicates of three from the unmodified control, Wood A, and Wood B. Under a vacuum, the sections were soaked in deionized water for 20 min and left submerged until the scan. Before the scan, the sections were wiped with tissue to remove the surface water. Naturally, this caused some variation in the WR values of different groups. This was done to reduce the otherwise dominant endothermic signal of the free water melting. Each section was placed in an aluminum hermetic pan (TA Instruments, #900793.901), which was sealed with a hermetic lid (#900794.901) using a crucible press. Different sections from the same block of wood were used. After the scan was completed, each pan was pierced with a tweezer and dried overnight at 105 °C to determine the dry wood mass. Three replicates were measured for each test group.

The water ratio of each sample was calculated using eq 1.
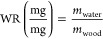
1where *m*_wood_ is the dry weight of the wood sample and *V*_water_ represents the volume of water absorbed during soaking.

### Differential Scanning Calorimetry

The bound and bulk water content was measured using a differential scanning calorimeter (TA Instruments, Q20) equipped with a cooling unit (RCS 90, Refrigerated Cooling Unit). The DSC calibration was performed using an indium standard (8 mg), which was heated through its melting transition at 156.60 °C at a heating rate of 10 °C/min. The ratio of the theoretical melting transition to the real value was used to calibrate the cell constant and the temperature. During the scan, each sample was stabilized at −30 °C to guarantee complete freezing of freezable water. Subsequently, the temperature was raised at a constant heating rate until the melting transition was completed, and the heat flow had returned to the baseline level. Each specimen was run consecutively at the heating rates 1, 5, and 10 °C/min. The melting transition of water-soaked wood displayed two overlapping endothermic peaks, corresponding to free water (Figure S3). The whole melting transition was integrated between −15 and 25 °C, using a sigmoidal baseline to account for height difference between baselines. The subpeaks were split at the local maximum, using a perpendicular drop separation (Figure S3).

The mass of free water was calculated according to eq 2, where *H*_m, free water_ is the melting enthalpy under the free water peak in the thermogram (Figure S3) and *H*_f_ is the latent heat of fusion of water (333.7 J/g). The mass of Type I water was calculated by subtraction of free water from the total amount of water in the sample in eq 3, where *m*_WS_ is the water-soaked mass of the sample and *m*_OD_ is the oven dry weight.
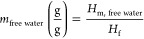
2
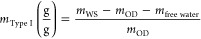
3

### Solid-State Nuclear Magnetic Resonance Spectroscopy and Relaxation Time Analysis

For relaxation time analysis, we selected samples of the unmodified control groups with a dry and ambient moisture content, a resin impregnated but uncured sample with an ambient moisture content, and dry heat cured specimens of Woods A and B that were not soaked in water. All samples were crushed to a fine powder using a laboratory microdismembrator.

Experiments were performed on a Bruker Avance III HD spectrometer using a 4 mm triple-resonance Bruker HXY probe that operated at a magic angle spinning (MAS) frequency of 12.5 kHz in double-resonance mode. The Larmor frequencies of the ^1^H and ^13^C nuclei were 500 and 125 MHz, respectively. Spectra of ^1^H and ^13^C were referenced indirectly to TMS using the ^1^H and ^13^C peaks of the external reference alanine. These peaks were at 1.1 and 20.5 ppm for ^1^H and ^13^C, respectively. A ^1^H 90° pulse with a duration of 2.5 μs was used, corresponding to a nutation frequency of 100 kHz. All experiments used a recycle delay of 3 to 4 s. Cross-polarization (CP) experiments were performed using a ramp of 70–100% on the ^1^H channel to meet the Hartman–Hahn condition, so as to transfer magnetization from ^1^H to ^13^C via ^1^H–^13^C dipolar couplings,^29^ with a contact time of 2 or 5 ms (apart from 1D ^13^C CP MAS experiments, where the contact time was 2 ms for all experiments). SPINAL-64 heteronuclear decoupling was performed during the acquisition of the ^13^C free induction decay.^30^

Spin–lattice relaxation time *T*_1_ (^1^H) was measured by using a saturation–recovery pulse sequence. A minimum of eight 90° pulses was applied until there was no observable signal, followed by a variable delay (τ) to allow for the sample to recover the signal. Subsequently a ^1^H 90° pulse was applied, and the signal intensity was measured. The relaxation time was calculated according to Frye.^31^

The spin–lattice relaxation *T*_1ρ_ was measured by using an initial ^1^H 90° pulse and subsequently a pulse for a duration of τ on the ^1^H channel. Finally, magnetization transfer to ^13^C was achieved, again using a ramp of 70–100% on the ^1^H channel to meet the Hartman–Hahn condition, and signal was detected on the ^13^C channel.

The *T*_1_ (^13^C) relaxation time was measured using the Torchia method.^32^ The experiment started off as a normal CP experiment. After the CP, a 90° pulse on the ^13^C channel rotates the magnetization from the transverse to the longitudinal plane. The nuclei then relax during the recovery time, τ. A second 90° pulse on the ^13^C channel then rotates the magnetization back into the transverse plane. This method measured *T*_1_ (^13^C) relaxation times up to 25 s directly, whereas higher values were interpolated based on plots of signal intensity against contact time. Long relaxation times were more prone to error; therefore, all *T*_1_ (^13^C) relaxation times of resin and most values for C1 (105 ppm) and C4 (89 ppm) were excluded. Similar issues have been reported previously in the literature.^8,25^

## Results

### Resin Treatment and ASE Tests

Table 1 summarizes the properties of Woods A and B after heat curing. The average liquid resin uptake (LU) after impregnation is 1.5 g in Wood A and 1.5 g in Wood B. The weight percentage gain (WPG) after heat curing ranges between 31.5% in Wood A and 32.6% in Wood B. Considering the standard deviation within each test group, it is evident that both Wood A and Wood B obtained an identical gravimetric resin uptake.

**Table 1:** Effect of Different Diffusion and Drying Conditions on the Performance of Wood A (Facilitated Cell Wall Diffusion) and Wood B (Restricted Cell Wall Diffusion) in ASE Tests during the First Water Soaking Cycle^a^

	LU in g		WPG in %		BU in %		*S*_1_ in %		*S*_2_ in %		*S*_3_ in %		ML_1_ in %		WU_1_ in %	
Wood A	1.5	(0.03)	31.5	(1.3)	9.7	(0.4)	9.7	(0.5)	11.9	(0.4)	12.4	(0.3)	2.7	(0.1)	98.3	(3.1)
Wood B	1.5	(0.03)	32.6	(0.7)	7.1	(0.3)	11.5	(0.2)	13.9	(0.4)	14.1	(0.7)	3.1	(0.2)	97.3	(2.0)

a Average values are shown with the standard deviation in parentheses. LU, liquid resin uptake; WPG, weight percentage gain; BU, bulking coefficient; *S*_1_, *S*_2_, and *S*_3_, swelling coefficient in different soaking cycles; ML_1_, mass loss after 1st cycle; WU_1_, water uptake.

The average bulking coefficient (BU) ranges between 9.7% in Wood A and 7.1% in Wood B. The swelling coefficients (*S*_1_, *S*_2_, and *S*_3_) in successive soaking cycles are 9.7%, 11.9%, and 12.4% in Wood A and 11.5%, 13.9%, and 14.1% in Wood B. Since the gravimetric resin uptake is the same, the observed differences in BU, *S*_1_, *S*_2_, and *S*_3_ are thought to be the result of different applied process conditions. The positive BU values in Table 1 suggest that cell wall diffusion took place in both groups, although it was more effective in Wood A than in Wood B. The BU of Wood A is 16% higher in Wood A and *S*_1_ is 27% lower than that in Wood B. In other words, Table 1 suggests that the resin molecules were able to penetrate the cell walls of Wood A better than those in Wood B. The mass loss during the first soaking cycle was also lower in Wood A, indicating better resin fixation. The mass loss in the successive cycles and the water uptake during each soak cycle show no difference (Table S1). Future work could also consider the effect of solvent extraction prior to the resin treatment.

### Scanning Electron Microscopy

**Figure 1:**
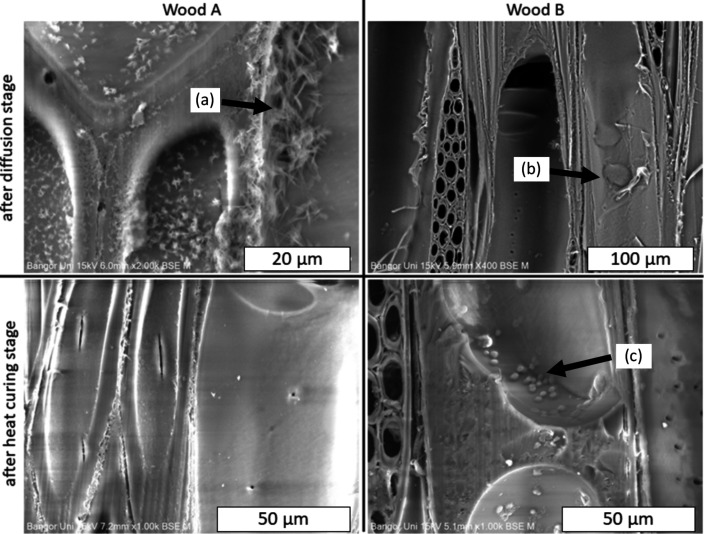
Scanning electron microscopy images of Wood A (facilitated cell wall diffusion) and Wood B (restricted cell wall diffusion) at different stages of the modification process. Views are on the tangential–longitudinal plane. Sections were prepared using a dry cut to avoid leaching of resin which might have occurred if samples were softened in water for a wet cut. (a) Resin “needles” or “cobwebs”, (b) resin hemispheres in uncured resin, and (c) resin spheres in cured resin.

Scanning electron micrographs (SEM) of Wood A and Wood B are shown in Figure 1. More images are provided in Figures S4 and S5. As was noted in previous studies, we observed resin coated lumens and resin filled lumens.^33,34^ There was no notable difference in the quantity of resin filled lumens. However, Figure 1 shows slightly different features in Woods A and B. After diffusion, the resin in Wood A forms needle-like structures that merge to larger cobweblike structures in the cell lumens. These resin “needles” or “cobwebs” disappeared after heat curing. The resin in Wood B agglomerates to a hemispherical shape after diffusion. After curing, these hemispheres detach and shrink, becoming spheres with a diameter of 1–5 μm. The number of these resin spheres in the lumen is much lower than in previous studies, where similar structures have been observed.^35^

While SEM cannot reveal the diffusion within the cell wall, the morphology of the resin deposits on the cell wall surfaces might hint toward the resin mobility. The higher humidity and temperature in treatment A improve the mobility of resin monomers during the diffusion stage; hence resin is dispersed across the macroscopic internal wood surface and delicate resin structures form in the lumens. Also, the moisture content in the diffusion stage remained high for longer in Wood A, hence the micropore network remained open for longer, permitting cell wall diffusion (Figure S6). Given enough time, cell wall diffusion might achieve an equilibrium state, where resin monomers are dispersed uniformly throughout the cell wall micropores. The hemispherical structures in Wood B, on the other hand, indicate that resin monomers might fail to overcome cohesive forces and tend to agglomerate. The moisture content in Wood B drops below 10% early in the diffusion process, resulting in the partial collapse of the micropore network (Figure S6).

### Differential Scanning Calorimetry

Differential scanning calorimetry (DSC) is a well-established method for elucidating certain aspects of the wood–water relationship. It uses the fact that bound water (Type I) in the cell wall does not undergo a phase transition like free water, which is located in the lumens. Thus, the method is frequently used to estimate the bound water content or fiber saturation point of wood and other natural polymers.^12,28,36−40^ In this study, the focus is on Type I water.

The water ratio (WR) in Table 2 ranges between 0.81–0.96 μL/mg, which is the result of differences in the capillary void volume available in modified and unmodified tulipwood.^27^ When the cell wall and the micropore network are fully saturated with water, any additional water ceases to affect the values of bound water; hence, any variation between samples is caused by the modification itself and not by differences in the WR. Park et al.^12^ have shown this for kraft pulp fibers, where Type I water ceased to change above a WR of 0.8 mg/mg, and the same is assumed for this experiment.

**Table 2:** Average Values of Water Ratio (WR) for Bound Water (Type I) Determined by the Dynamic DSC Method Using Heating Rates (HRs) of 1, 5, and 10 °C/min^a^

	HR in °C/min	WR in mg/mg		*m*_Type I_ in g/g	
Control	1	0.96	(0.11)	0.386	(0.045)
A	1	0.81	(0.16)	0.189	(0.064)
B	1	0.89	(0.17)	0.241	(0.064)
Control	5	0.96	(0.11)	0.374	(0.045)
A	5	0.81	(0.16)	0.172	(0.046)
B	5	0.89	(0.17)	0.221	(0.065)
Control	10	0.96	(0.11)	0.363	(0.045)
A	10	0.81	(0.16)	0.170	(0.047)
B	10	0.89	(0.17)	0.216	(0.065)

a Standard deviation in parentheses. Wood A (facilitated cell wall diffusion) and Wood B (restricted cell wall diffusion).

Table 2 shows the Type I water content for the unmodified control and heat cured samples of Wood A and Wood B at different heating rates. Depending on the heating rate, the measured Type I water content of the control ranges between 0.363–0.386 g/g, which corresponds well with the values reported in the literature. Thybring and Fredriksson^40^ report values between 0.250–0.440 g/g for the Type I water content in various unmodified wood species.

Depending on the heating rate, the Type I water was reduced to 0.170–0.189 g/g in Wood A and to 0.216–0.241 g/g in Wood B. Hence, a substantial reduction is observed for both treatments, but the reduction in Type I water was greater in Wood A (i.e., 51% reduction at 1 °C/min) compared to Wood B (i.e., 38% reduction at 1 °C/min). It is reasonable to assume that micropore blocking limits Type I water uptake in the modified timbers. Thus, microscopic resin deposits in the cell wall are lowering the bound water content through spatial confinement,^40^ and Table 2 shows that this micropore blocking effect is more effective in Wood A compared to Wood B.

Therefore, it can be summarized at this point that both cell wall bulking (Table 1) and micropore blocking (Table 2) are more effective in Wood A than in Wood B, despite the similar gravimetric resin content. This is clearly related to differences in cell wall diffusion during the different treatment processes. These differences are investigated in the following NMR section.

### Solid-State Nuclear Magnetic Resonance Spectroscopy

**Figure 2:**
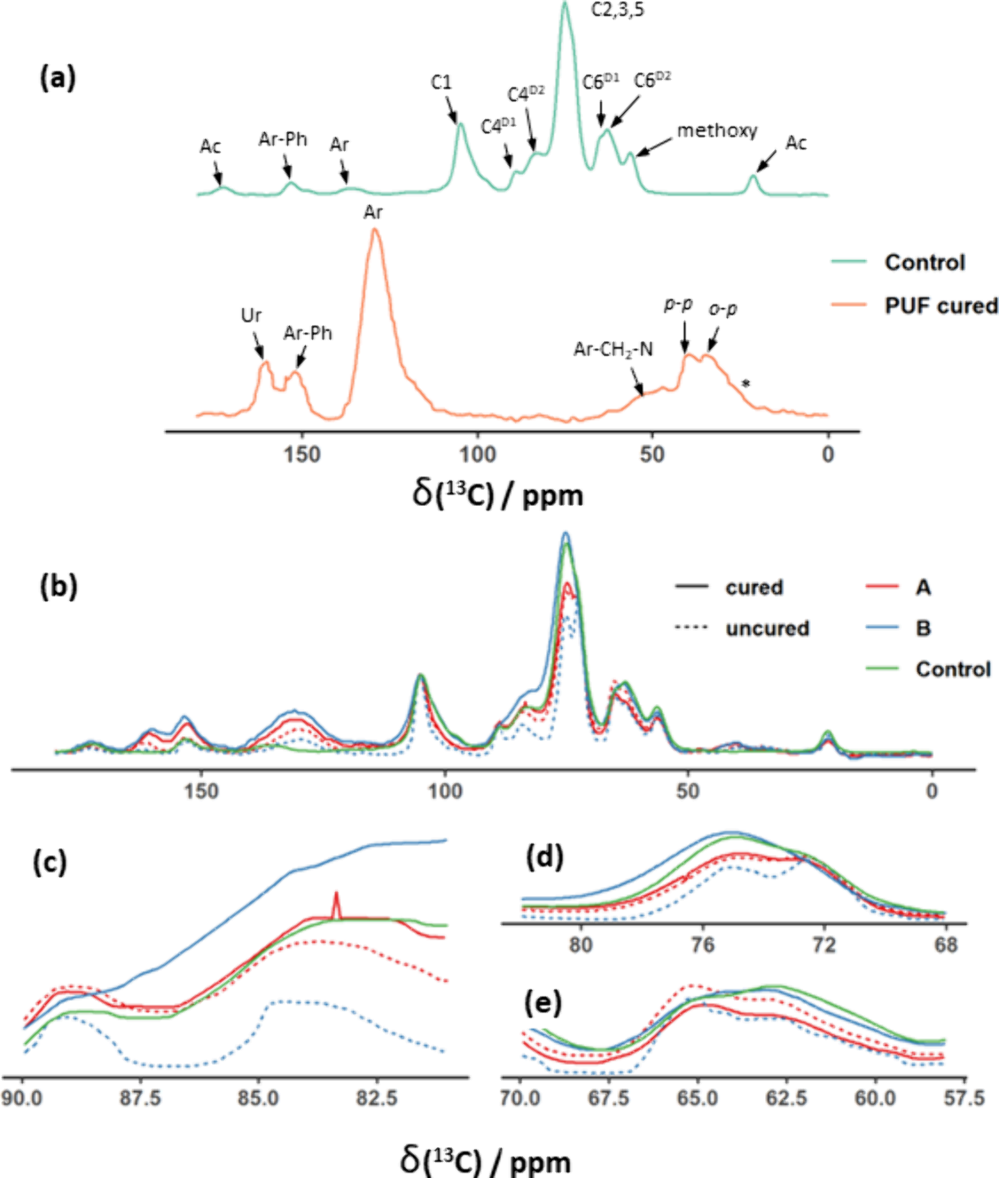
^1^H (500 MHz) ^13^C CP MAS (12.5 kHz) NMR spectra of (a) unmodified dry tulipwood and pure heat cured PUF resin with peak assignments. Ac indicates the O–acetyl group in hemicellulose, C1 to C6 indicate cellulose ring positions in domain 1 (D1) or 2 (D2), Ar indicates aromatic ring carbons, Ar-Ph indicates phenoxy carbons, *o-p* and *p-p* indicate methylene bridges in an *ortho–para* and *para–para* constellation, respectively, Ar–CH_2_–N indicates methylene bridges of co-condensed urea and phenol, and Ur indicates the urea carbonyl signal. (b) Comparison of uncured and cured Wood A (facilitated cell wall diffusion) and Wood B (restricted cell wall diffusion) and (c–e) spectral regions for unmodified tulipwood, Wood A, and Wood B before and after heat curing. Dashed lines are for spectra of resin impregnated but uncured samples. Plain lines are for spectra of heat cured wood samples. (c) Close-up of the C4 signal which is divided into a C4^D1^ (89 ppm) and C4^D2^ signal (84 ppm), (d) close-up of the C2, C3, and C5 signal, and (e) close-up of the C6 signal, which is divided into a C6^D1^ (66 ppm) and C6^D2^ (62 ppm) signal. A spinning sideband in the spectrum of the PUF resin is marked with an asterisk.

Figure 2a shows ^13^C CP MAS NMR spectra of the unmodified wood (control) and heat cured PUF resin. Note that the peak intensity in such CP MAS NMR spectra depends on the magnitude of ^13^C–^1^H dipolar couplings by which magnetization is transferred from ^1^H to ^13^C. As such, peak intensity does not depend quantitatively only on the number of carbon atoms, but rather is modified by molecular mobility as well as whether there are directly bonded hydrogen atoms. Therefore, care must be taken with interpretation of peak intensities; however, it is meaningful to comment on changes between two spectra, e.g., before and after curing, and differences in intensities between moieties with the same atomic connectivities, e.g., domain 1 and domain 2 cellulose. Peaks in the unmodified control are assigned as follows. The anomeric carbon bearing the glycosidic bond (C1) is shown at 106 ppm. The other glycosidic carbon (C4) is observed at lower ppm values and can be separated into C4^D1^ (89 ppm) and C4^D2^ (84 ppm) signals corresponding to cellulose domains 1 and 2.

Similarly, exocyclic carbon (C6) in cellulose shows a C6^D1^ (66 ppm) and a C6^D2^ (62 ppm) signal. The large peak at 75 ppm corresponds to the overlapping of three different ring positions (C2, C3, and C5) in cellulose as well as the majority of hemicellulose carbon atoms. The lignin methoxy group shows a distinct peak at 57 ppm. Lignin aromatic signals are observed at 136 ppm with low intensity. Quaternary lignin carbons bearing the phenoxy group are shown at 153 ppm. Xylan, which is the only acetylated hemicellulose in hardwoods,^41^ is represented by the methyl and the carbonyl signal of the acetate group at 21 and 172 ppm, respectively.^25,26^

The cured PUF resin shows a major peak at 130 ppm, which is assigned to all phenolic ring positions. Phenoxy carbons are at 152 ppm and urea carbonyls at 161 ppm.^42,43^ Methylene bridges (*o-p*, *p-p*) of self-condensed phenolic cores display an intense double peak between 40 and 34 ppm. Methylene bridges of co-condensed phenolic and urea structures show up as a shoulder between 55 and 45 ppm.^42^ Hydroxymethyl groups, which cross-link during heat curing, are depleted in the cured resin, and show only a small shoulder at 61 ppm.

The spectra of unmodified wood and resin barely overlap, which is important for the analysis of relaxation times in the following section. Hence, it is reasonable to assume that in modified wood, the region between 105 and 65 ppm corresponds exclusively to wood carbohydrates, the region between 160 and 120 ppm and the peak at 40 ppm to PUF resin, and the peaks at 21 and 172 ppm to xylan. There is some degree of overlapping in the region of 65–50 ppm, which may affect the analysis of the C6 and methoxy carbons. Figure 2b shows that there are no new peaks present in the modified wood, suggesting the absence of new covalent bonds between resin and wood.^25^

The effect of resin impregnation and heat curing can be observed in Figure 2b. Compared to the control samples, the presence of uncured resin causes an increase in the resin specific peaks at 161, 152, 130, and 40–30 ppm. Figures 2c and 2e show close ups of peaks corresponding to the C4 and C6 regions, respectively. Resin impregnation (without curing) reduces the C4^D2^ and C6^D2^ signals compared to their domain 1 counterparts and leads to peak narrowing, which indicates an increase in the mobility or a more organized structure. The peak at 74 ppm for C2, C3, and C5 is shown in Figure 2d. The single peak present in unmodified wood splits into a double peak in the presence of uncured resin.

Comparing the full line (cured) and dashed lines (uncured) in Figure 2b, it is evident that, after curing, resin signals at 161, 152, and 130 ppm were more intense in comparison to peaks in the carbohydrate region. This likely indicates a change in the molecular mobility of the resin when its monomers polymerize on curing. The partial degradation of carbohydrates during curing might also have an effect on peak height ratios. Figures 2b–2e show the distinct behavior of Wood A after heat curing. The cellulose domain 2 signals maintain a lower intensity compared to their domain 1 equiv in unmodified wood. Also, the C2, C3, and C5 peak remains split in Wood A (see Figure 2d, red solid line). On the other hand, cured Wood B resembles the unmodified control in the carbohydrate regions of the spectrum (zoomed regions as shown in Figures 2d and 2e). Significant peak broadening of the C4 and C6 signals can be observed in cured Wood B to an extent that domains 1 and 2 signals can no longer be distinguished. This indicates a decrease in mobility or a less organized structure.

The relative ratio of peak areas in the C4 region is frequently used to study the morphology of carbohydrates in wood.^44−46^ Usually, this is done after separating the subspectrum of cellulose from those of lignin and hemicelluloses based on different *T*_1ρ_ time constants of these components.^44^ In this study, no subspectra were obtained; therefore, we acknowledge some degree of overlapping between hemicelluloses and cellulose in the region of 84–80 ppm.^45^ Given that the peak intensity depends on the molecular mobility of the components, the ratio of cellulose domains D1:D2 must be interpreted cautiously in this study. Nevertheless, we choose to include the ratio D1:D2 in Table S2 because it illustrates interesting differences between Woods A and B. Previous studies observed an increasing ratio after resin impregnation but did not elaborate further.^26^ This increase corresponds to observations in the current study, where the presence of uncured resin increases the ratio significantly more in Wood A as compared to in Wood B. After curing, Wood A maintains a higher ratio than the dry control. In Wood B, the ratio decreases below the value of the dry control. Although it is difficult to extract clear information from this ratio, the opposite behavior of Woods A and B indicates differences in the interaction of resin with the microfibrils and cell wall nanopores. The unmodified control shows that the ratio increases upon water absorption in ambient conditions, which is in contrast to previous studies that investigated a softwood.^47^

## Solid-State Nuclear Magnetic Relaxation Time Analysis

### *T*_1_ (^1^H) Relaxation Times

Table 3 reports *T*_1_ (^1^H) relaxation times measured by an inversion recovery experiment, which correspond to slow molecular motions of protons in the kHz range. The relaxation times in Table 3 are derived from the broad peak at 3.2 ppm in the ^1^H MAS NMR spectrum (Figure S7). Consequently, *T*_1_ (^1^H) provides an average for all protons in a sample and is not specific to any functional group. The plots of signal intensity against contact time generally showed very close fit to a *T*_1_ build-up curve, as shown in Figure S8.

**Table 3:** Proton Spin–Lattice Relaxation Times *T*_1_ (^1^H) and the Ratio of Integrated Areas under the Peaks Representing the Cellulose Domain 1 (89 ppm) and Domain 2 (84 ppm) Signals in Wood^a^

sample	state	MC^b^ in %	*T*_1_ (^1^H) in s	
Control (dry)	unmodified	0.0	2.1	(0.06)
Control (ambient)	unmodified	7.5	0.8	(0.02)
PUF	cured	0.0	1.2	(0.02)
A	cured	0.0	3.1	(0.16)
A	uncured	7.9	2.7	(0.11)
B	cured	0.0	1.3	(0.04)
B	uncured	10.33	2.6	(0.14)

a MC is the gravimetrical moisture content before the measurement.

b Given the homogeneity of the powdered sample, we deemed a single measurement sufficient, which is why standard deviations for these data cannot be provided.

Previous studies have shown that *T*_1_ (^1^H) of cellulose and lignin in wood decreases from approximately 1 s in dry conditions to less than 0.2 s at the fiber saturation point.^24^ This is because water molecules that are absorbed to the biopolymer surface transfer magnetization between water and the biopolymer through “spin–spin flip flop exchange”.^24,44^ In dry conditions, however, the *T*_1_ (^1^H) relaxation occurs preferentially via the lignin methoxy group.^26^ This corresponds well with our results for *T*_1_ (^1^H) in unmodified wood, where the presence of just 7.48% water lowers the relaxation time considerably from 2.1 to 0.8 s (see Table 3).

Cured PUF resin (1.2 s) has a lower *T*_1_ (^1^H) time than dry unmodified wood (2.1 s), indicating that protons in the resin are more mobile on average. Since this is not necessarily expected, the faster relaxation in the resin may be driven by a single mobile functional group that is lowering the average. In the presence of uncured resin, *T*_1_ (^1^H) increases beyond the level of dry wood (2.1 s) and pure resin (1.2 s).

The *T*_1_ (^1^H) relaxation times of uncured Wood A (2.7 s) and Wood B (2.6 s) are similar at this stage of the process (i.e., uncured state). This increase compared to the control sample is evident despite an ambient moisture content, suggesting that the relaxation mechanism via water is hindered in both treatments. A similar trend was observed in previous studies where the *T*_1_ (^1^H) relaxation time of Japanese cypress wood increased from 1.2 to 2.1 s after PF resin impregnation.^25^ Variations to literature values are likely caused by different resin formulations and the different chemical wood compositions of hardwoods and softwoods. To restrict the relaxation via water, the resin must be in close contact with the microfibril and disturb the formation of hydrogen bonds with the tightly bound water. Previous work has shown that the *T*_1_ (^1^H) relaxation time of isolated cellulose increases with its potential to form hydrogen bonds as a function of the pH value.^48^ Therefore, hydrogen bonding between uncured resin and carbohydrates could also play a role in the increased *T*_1_ (^1^H) relaxation time of uncured Wood A and Wood B.

After heat curing, Woods A and B act in diametrically opposite ways. The *T*_1_(^1^H) time of Wood A increased from 2.7 s before curing to 3.1 s after curing. An increase in *T*_1_ (^1^H) time upon curing has not been previously observed and suggests that *in situ* resin curing restricts proton mobility in Wood A. The *T*_1_(^1^H) time of Wood B decreases from 2.6 s before curing to 1.3 s after curing, which is faster than the *T*_1_(^1^H) time for our control of dry unmodified wood. The decrease in the *T*_1_ (^1^H) time of Wood B upon curing is known in the literature^26^ and is a result of the averaging of the *T*_1_ (^1^H) relaxation times of dry unmodified wood and pure resin. By contrast, in Wood A, the interaction between wood and resin leads to an increased relaxation time, beyond the level of both individual components. This suggests that the resin and wood create a nanoscale composite at a scale of >30 nm, which has different properties to the individual components. In Wood B, the resultant average of wood and resin *T*_1_ (^1^H) relaxation times indicates that the resin and wood might coexist at a nanoscale but do not interact as substantially as in Wood A.

### *T*_1ρ_ Relaxation Times

**Figure 3:**
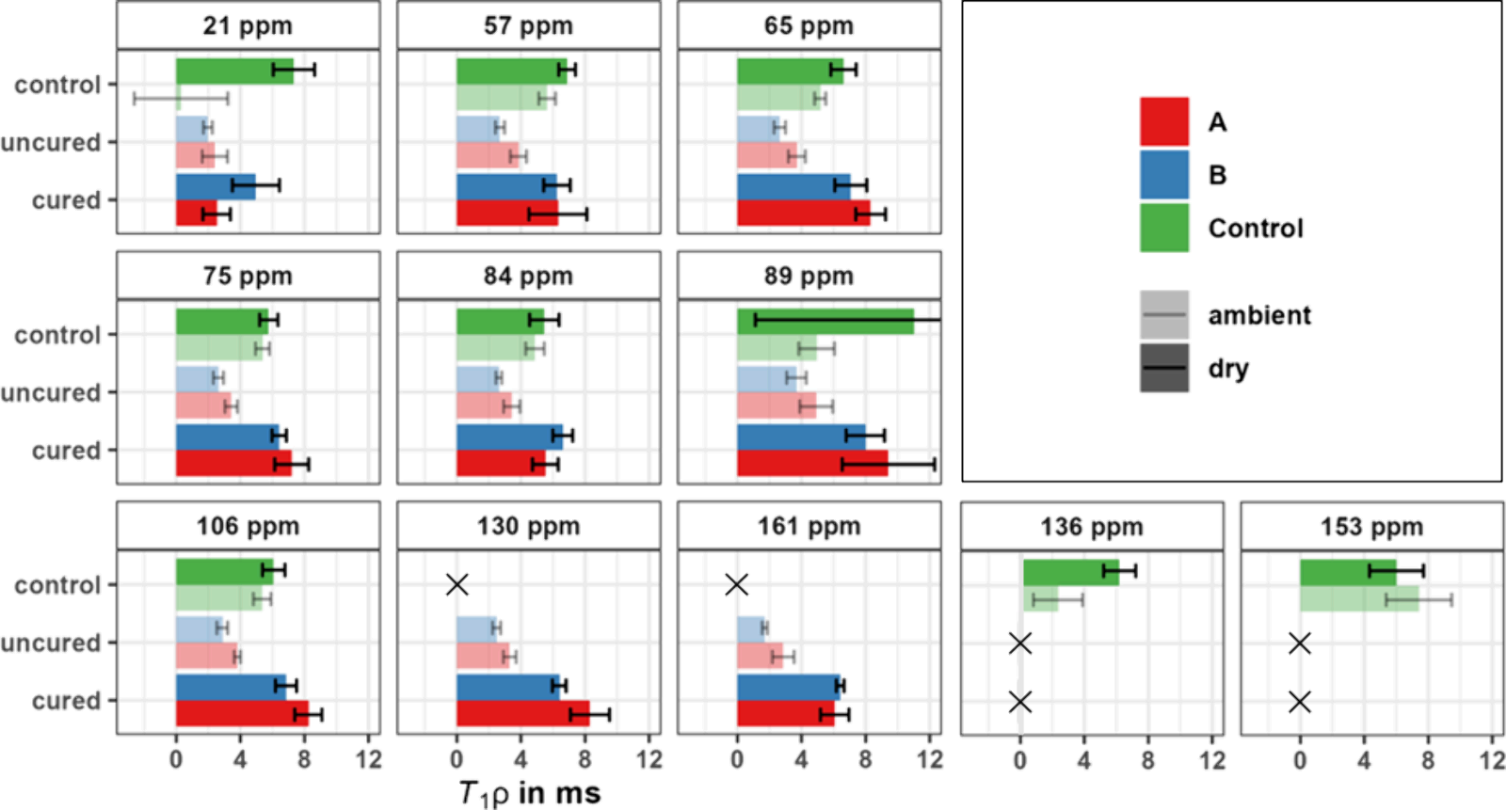
^1^H (500 MHz) *T*_1ρ_ of unmodified control, uncured, and cured samples of Wood A (facilitated cell wall diffusion) and Wood B (restricted cell wall diffusion). Each subplot shows *T*_1ρ_ for the resolved ^13^C peaks in the ^13^C CP MAS NMR spectrum (see Figure 2). The unmodified control was tested at two moisture contents, ambient and dry, to account for the effect of water on the cell wall. All uncured samples were tested at ambient conditions, and all cured samples were tested at dry conditions. The chemical shift at 21 ppm corresponds to the hemicellulose methyl acetate group; 57 ppm represents the lignin methoxy group. The chemical shifts of 106, 89, 84, 75, and 65 ppm correspond to cellulose and hemicelluloses, and the chemical shifts of 161 and 130 ppm are for PUF resin. For the sake of better illustration, the lignin signals of unmodified wood, which occur at 136 and 153 ppm, are displayed separately from the aromatic resin signal at 130 ppm and the urea carbonyl signal at 161 ppm in modified wood. After resin modification, these signals cannot be separated.

Figure 3 shows ^1^H *T*_1ρ_ relaxation times measured via a ^1^H–^13^C CP MAS NMR experiment. Each value in the figure corresponds to a peak in the ^13^C spectrum and is specific to a certain functional group. *T*_1ρ_ corresponds to the fast molecular motions of protons in the MHz range. The numerical values corresponding to the graphical representation in Figure 3 are listed in Table S3 and example data fits are shown in Figure S9. Note that we have only measured ^1^H *T*_1ρ_ relaxation times at one temperature, namely, room temperature for the input MAS gases; as such, care must be taken with an interpretation as to whether an increase or decrease in the relaxation time is associated with an increase or decrease in mobility.

Previous studies on unmodified wood with a moisture content of 35% show that the *T*_1ρ_ relaxation times of hemicelluloses and lignin are highly correlated suggesting compatibility on a nanoscale, while the *T*_1ρ_ relaxation of cellulose is typically longer, both in dry and wet conditions.^21,24^ Newman measured a *T*_1ρ_ relaxation time of 7.2 ms at 89 ppm and 3.9 ms at 56 ppm in wet Radiata pine samples.^24^ The same study noted that the *T*_1ρ_ relaxation mechanism via water is more significant in lignin and hemicelluloses than in cellulose. Most of these observations about unmodified wood agree well with our results in Figure 3, however, the methoxy signal at 57 ppm is similar to the cellulose signals in both dry and ambient conditions, which differs from Newman’s observations. These differences may be attributed to variations in moisture content (35% in Newman’s study vs 7.5% in the current) and the different wood species used (*Eucalyptus delegatensis* vs *Liriodendron tulipifera*).

The *T*_1ρ_ relaxation times in the dry control are consistently between 6 and 7 ms (except at 89 ppm where there is a very large error bar), indicating that cellulose, lignin, and hemicellulose are spin-coupled in dry conditions. Changing the moisture content from dry to ambient conditions has two effects on the unmodified control. For one, *T*_1ρ_ decreases in all parts of the spectrum except for 153 ppm. In the case of the C2, C3, and C5 signals at 75 ppm and the aromatic lignin signal at 136 ppm, a variable-temperature study has established that the decrease in *T*_1ρ_ relaxation times correlates with faster molecular motions.^25^ However, we are not aware of this relationship, having been previously investigated for the other carbons in the spectrum, and thus, as noted above, statements about the molecular mobility of the remaining carbons are not made.

Slower *T*_1ρ_ relaxation times for the cellulose signals (105–60 ppm) and faster times for the lignin and xylan signals (136 and 21 ppm), which display a more pronounced decrease in the presence of water,^21,24^ indicate a second key feature, namely phase separation. This phase separation in the ambient moisture state corresponds well with a more recent model of the hydrated cell wall, where the adsorption of water disturbs the lignin-hemicelluloses and cellulose interface.^47^

In the presence of uncured resin, *T*_1ρ_ values in the carbohydrate region (105–60 ppm) decrease compared to the ambient untreated control; hence, uncured resin enhances the fast molecular motions of the C2, C3, and C5 carbons.^25^ This is in contrast to the slow molecular motions, represented by *T*_1_ (^1^H), which are restricted (see Table 3). The same trend was observed by Nishida et al., although in their study the *T*_1ρ_ relaxation time of the 75 ppm signal, for both modified and unmodified wood, was generally longer than in the present study at 10–17 ms.^25^ In uncured Wood A, the *T*_1ρ_ relaxation times of lignin (57 ppm), cellulose C1, C2, C3, C4^D2^, and resin (130 ppm) are all in the range of 3–4 ms, which could be caused by spin diffusion through close spatial proximity. The hemicellulose methyl acetate (21 ppm) in uncured Wood A has a faster *T*_1ρ_ relaxation time (2.5 ms) than the other cell wall constituents, as it is a side chain, which will always be more mobile than the polymer backbone. The relaxation pathway via water might still be effective in the hemicelluloses of uncured Wood A. The *T*_1ρ_ relaxation time of cellulose C4^D1^ is notably slower than the rest of the sample, with 4.9 ms. Hence, with a difference of 1.5 ms between the *T*_1ρ_ relaxation times for the ^13^C chemical shifts at 84 and 89 ppm, it is likely that the two cellulose domains are not spin-coupled, which would align with the observations from Laborie.^8^ The trends in uncured Wood B are similar to those of Wood A, with the only difference being that the *T*_1ρ_ relaxation times are typically 0.5–1 ms shorter than those in uncured Wood A (see Table S3).

After heat curing, the *T*_1ρ_ relaxation time increases to a level similar to or slightly beyond that of unmodified dry wood in most parts of the spectrum. The literature shows that *T*_1ρ_ of pure resin increases with the degree of cross-linking as the molecular mobility decreases, which is consistent with the longer *T*_1ρ_ values of cured wood compared to uncured wood at 130 ppm.^49^ In cured Wood A, cellulose C1, C2, C3, and C5 and resin (130 ppm) have a similar *T*_1ρ_ relaxation time between 7.2 and 8.3 ms. Slightly shorter *T*_1ρ_ relaxation times between 5.5–6.3 ms suggest that lignin (57 ppm), cellulose C4^D2^, and the resin carbonyl (161 ppm) constitute a separate phase. Cellulose C4^D1^ has a notably longer *T*_1ρ_ relaxation time of 9.2 ms, which indicates that the phase separation between domain 1 and 2 persists after cure; however, the standard error for Wood A is relatively high. Surprisingly, after curing Wood A, the *T*_1ρ_ relaxation time of hemicellulose is still as low as in the uncured state, only 2.5 ms. Despite the decreased *T*_1ρ_ relaxation time of the methyl acetate group, due to being a side chain on the xylan, it is clear that the *T*_1ρ_ relaxation time is affected differently in Wood A and Wood B.

The trends upon curing in Wood B are slightly different, mainly because the *T*_1ρ_ relaxation times of all components are in a much closer range. Except for hemicellulose and C4^D1^, all *T*_1ρ_ relaxation times are between 6.2–7.1 ms. The hemicellulose fraction has a *T*_1ρ_ relaxation time of 5 ms and is therefore slightly slower than that in Wood A. The *T*_1ρ_ relaxation time of C4^D1^ is 8 ms in cured Wood B, which is slower compared to that in cured Wood A, though the difference is within the error bars.

### *T*_1_ (^13^C) Relaxation Times

**Figure 4:**
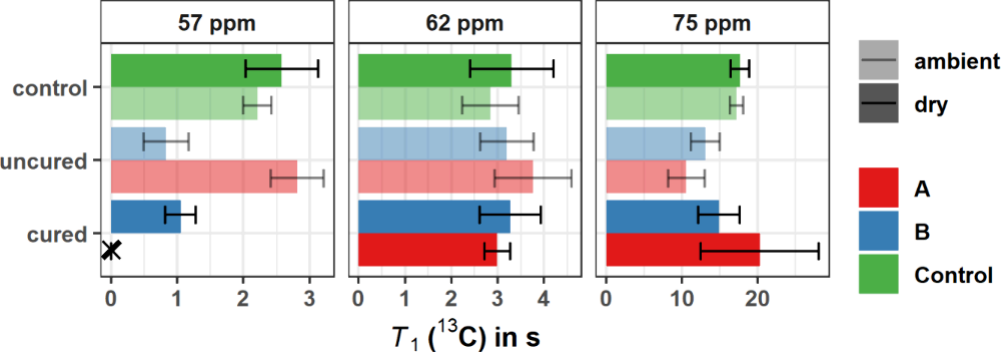
^13^C (125 MHz) *T*_1_ (^13^C) of unmodified control, uncured, and cured samples of Wood A (facilitated cell wall diffusion) and Wood B (restricted cell wall diffusion). Each subplot shows *T*_1_ (^13^C) for the resolved ^13^C peak in the ^13^C CP MAS NMR spectrum (see Figure 3). The unmodified control was tested at two moisture contents, ambient and dry, to account for the effect of water in the cell wall. All uncured samples were tested at ambient conditions and all cured samples were tested at dry conditions. The chemical shift at 57 ppm corresponds to the lignin methoxy group, and chemical shifts of 62 and 75 ppm correspond to cellulose and hemicelluloses. Chemical shifts between 160 and 105 ppm did not give credible results, i.e., with very large fitting errors, and are therefore not included. The missing value for dry Wood A at 57 ppm is indicated by an X symbol.

The *T*_1_ (^13^C) relaxation times were measured by ^13^C CP MAS NMR using the Torchia method.^32^ For our samples at natural abundance, these values are specific to specific functional groups and represent slow molecular motions (kHz range). Figure 4 shows the *T*_1_ (^13^C) relaxation times for different ^13^C chemical shifts, with Table S4 listing the numerical values and Figure S10 presenting representative data fits.

Previous studies on unmodified wood have shown that *T*_1_ (^13^C) relaxation times of cellulose and hemicellulose decrease with increasing moisture content.^21,25^ In the case of hemicelluloses, where *T*_1_ (^13^C) is more sensitive to the presence of water, this can be explained by a moisture induced glass transition that occurs at a moisture content of around 15%.^18^ The trend from the literature is partly confirmed in our study as the *T*_1_ (^13^C) relaxation times for the lignin methoxy group (57 ppm) and the cellulose positions C2, C3, C5, and C6 decrease slightly for the ambient samples. The *T*_1_ (^13^C) relaxation times of the other signals in the spectrum displayed a high error, which is similar to other studies.^8,25,26^

Previous literature suggests that the presence of uncured resin prolongs the *T*_1_ (^13^C) relaxation time at 74 ppm, whereas absorbed water has an accelerating effect.^25^ Nishida et al. measured a *T*_1_ (^13^C) relaxation time of 28–35 s at 74 ppm for unmodified wood at different moisture contents. They showed that the presence of uncured resin (20% w/w) caused an increase to 53 s when measured in dry conditions; however, in the ambient uncured state, the *T*_1_ (^13^C) was 34 s. Hence, the effects of water and uncured resin on the *T*_1_ (^13^C) relaxation time at 74 ppm counterbalance each other. Figure 4 shows that the *T*_1_ (^13^C) relaxation time at 74 ppm is generally shorter in uncured wood as compared to that in unmodified wood. The effect of moisture at 74 ppm seems to be minor, as the *T*_1_ (^13^C) relaxation time is the same value for the ambient and dry control group. In contrast to previous work, uncured resin seems to accelerate *T*_1_ (^13^C) for C2, C3, and C5. Concerning the *T*_1_ (^13^C) relaxation time for C6, the literature suggest that this value should be in a similar range to the lignin methoxy group and that the *T*_1_ (^13^C) relaxation times for both C6 and methoxy are 1 order of magnitude faster than that for C2, C3, and C5. This is confirmed for this study, but the high deviation for the C6 signal allows no further discussion of trends. An interesting trend is observed in the lignin methoxy group. In uncured Wood B, the *T*_1_ (^13^C) relaxation time at 57 ppm decreases from 2.2–2.6 s in the controls to 0.8 s in the presence of uncured resin. This decrease is not observed in Wood A and indicates that resin in Wood B is more associated with the lignin fraction at a scale of >30 nm. This could be within either the secondary cell wall or the middle lamella. After heat curing, the *T*_1_ (^13^C) relaxation time of lignin in Wood B remains lower than that observed for the control samples (1.1 s). Further statements about the effect of heat curing on *T*_1_ (^13^C) cannot be made due to the high errors in the *T*_1_ (^13^C) relaxation time data fits.

## Discussion of Solid-State NMR Data

**Figure 5:**
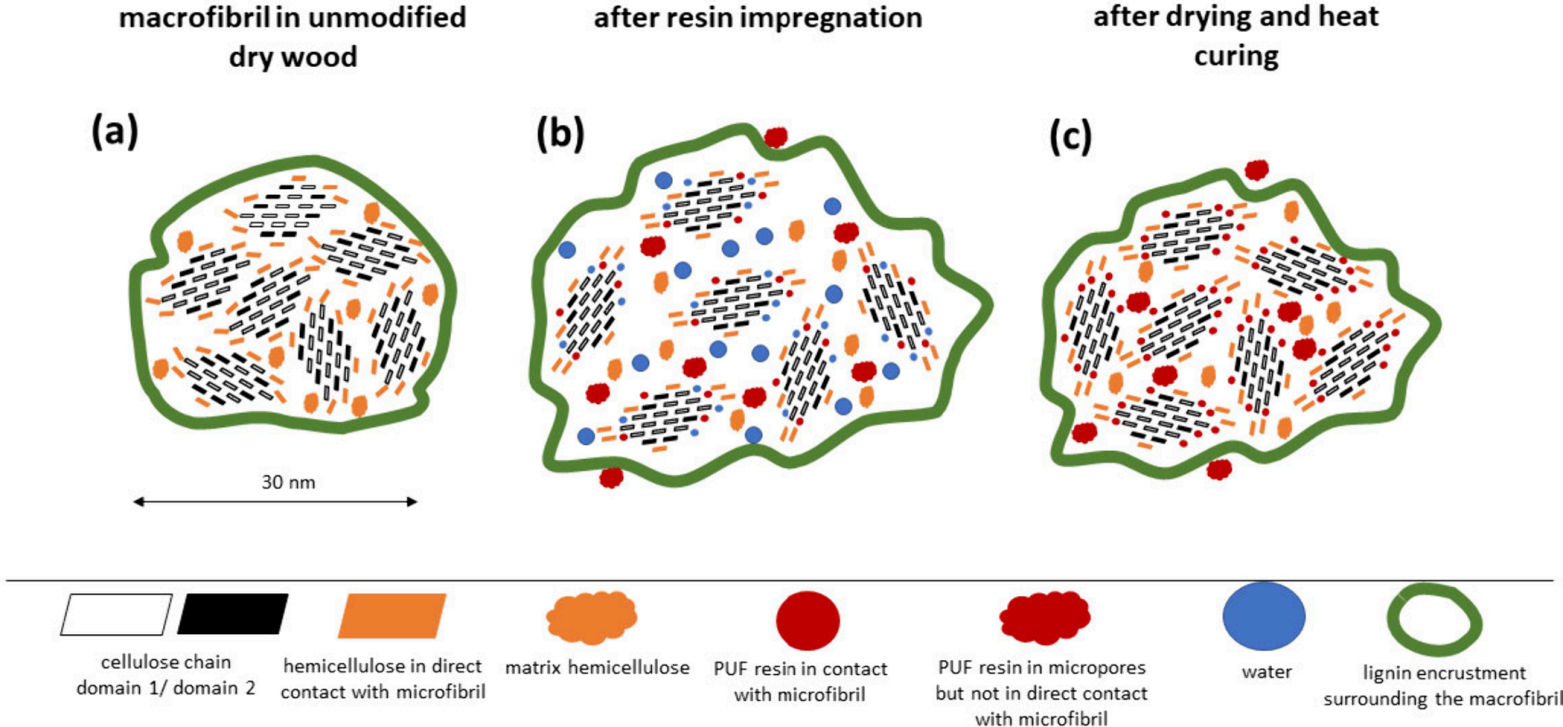
(a) The macrofibril model in different stages of the resin treatment. In the dry unmodified state, the macrofibril and microfibrils are illustrated according to recent literature.^47,50^ (b) Upon resin impregnation, both water and resin molecules create micropore spaces between the microfibrils. The Type I water fraction is closely associated with the microfibril. Similarly, it is proposed that resins occur in at least two distinct populations. Resin in direct contact with the microfibril disturbs hydrogen bonding between water and cellulose, causing *T*_1_ (^1^H) to increase and the Type I water content to decrease. The disruption of hydrogen bonding at the microfibril interface might cause some cellulose chains to change from a domain 2 conformation to domain 1, explaining the increased D1:D2 ratio. The other fraction of resin might be located in the cell wall but not in direct contact with the cellulose–hemicelluloses interface. The second resin population might be more closely associated with matrix hemicelluloses and lignin. (c) After heat curing, the resin induces a cell wall bulking effect.

**Figure 6:**
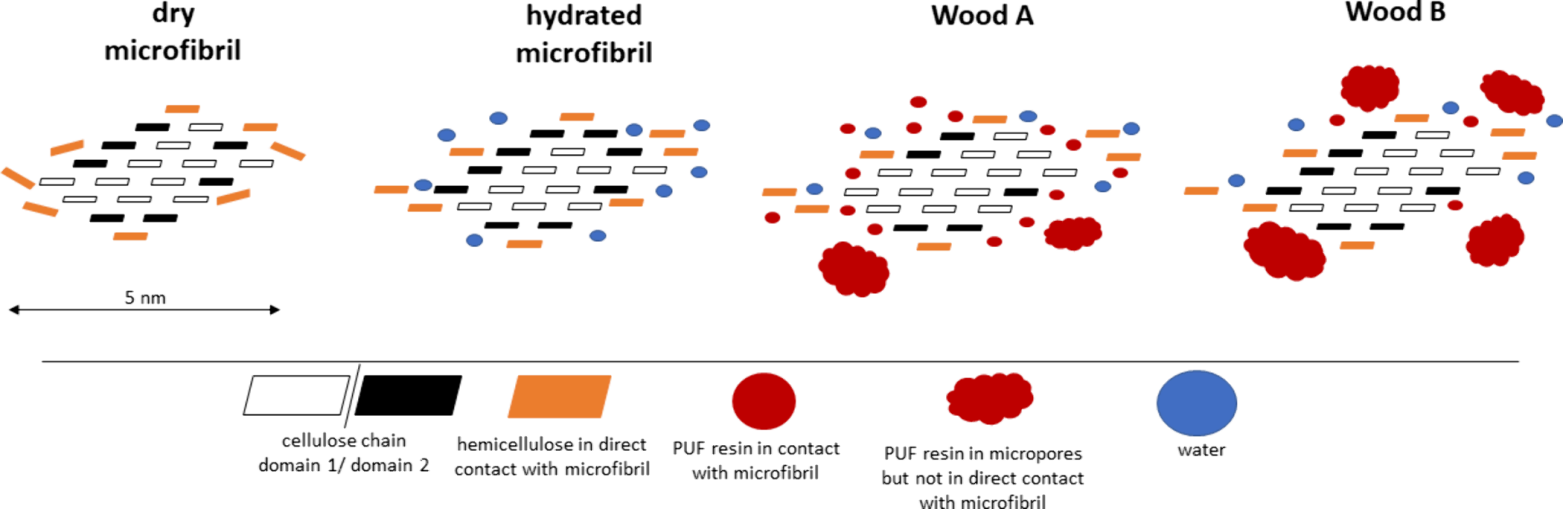
Model of microfibrils. The main difference between Wood A (facilitated cell wall diffusion) and Wood B (restricted cell wall diffusion) could be the relative content of both resin populations, where Wood A contains more resin that is in direct contact with the microfibril and Wood B contains more resin of the second population that exhibits greater association with matrix hemicelluloses and lignin. Atmospheric conditions applied to Wood A have promoted diffusion to the microfirbil surface and subsequent curing in this location. Conditions applied to Wood B have potentially favored resin agglomerations in the cell wall.

Based on our observations, we propose a model regarding wood–resin interactions, which is illustrated in Figures 5 and 6. The macrofibril is presented as previously described by Terrett et al.^50^ containing several microfibrils with a 2–3–4–4–3–2 arrangement of cellulose chains. Parts of the hemicelluloses are directly absorbed to the microfibril surface via hydrogen bonding, while other parts exist more independently, forming a matrix between microfibrils. Lignin encrusts the macrofibril and is in direct contact with some of the bound hemicelluloses. This model has been extended by Cresswell et al.^47^ for the hydrated cell wall, where adsorbed water is integrated in the structure of the microfibril. Water adsorption disturbs the lignin-hemicellulose and cellulose interface. Hydrogen bonding between water and the surface of the microfibril changes the C4^D1^ conformation to C4^D2^ in some cellulose chains.

Resin penetrates the interface between cellulose and hemicellulose, where it breaks existing hydrogen bonds and deacetylates parts of the hemicelluloses, as described by Nishida et al.^25^ It is known from the literature that a disproportionately high concentration of bound water in the cell wall is found at the interface of cellulose and hemicellulose, and that this interface plays an important role in the adsorption of water as well as in the swelling and shrinking of the cell wall.^51,52^

In our model, we distinguish two fractions of resin in the cell wall. It is proposed that one resin fraction is located directly at the interface between cellulose and hemicellulose. The second resin fraction is also located inside the cell wall but is not in direct contact with the microfibril surface. It is assumed that the atmospheric conditions applied to Wood A have favored the diffusion of resin to the interface region. In Wood B, however, diffusion conditions have probably disfavored resin diffusion to the interface region, leading to a higher proportion of the second resin fraction.

The previous studies have considered softwood cell walls, which have a different chemistry than the hardwood cell wall in terms of hemicelluloses and lignin composition.^41,53^ This is of relevance here because it may influence the current study where the hardwood species tulipwood was used. Nevertheless, our results for unmodified wood confirm many aspects of previous studies. In the dry control group, all cell wall polymers are in the same morphological phase of 2–30 nm, as suggested by a common *T*_1ρ_ relaxation time between 6–7 ms. Water adsorption in the control leads to a phase separation, as the mobile lignin-hemicellulose phase is pushed away from the more rigid cellulose phase. The cellulose domains 1 and 2 have a common *T*_1ρ_ relaxation time in ambient conditions and are therefore in the same morphological phase. In ambient conditions, water absorbed to the microfibril transfers magnetization via spin–spin relaxation and thereby decreases the *T*_1_ (^1^H) significantly compared to the dry state. The decrease of *T*_1_ (^13^C) at 21 ppm indicates a softening of hemicelluloses in ambient conditions (Table S4).

Both modified timbers display a very different behavior as compared to the control group, indicating various changes in the molecular architecture of the cell wall. In uncured Wood A, we observed peak narrowing of both C4 signals in the MAS NMR spectra, indicating an increase in mobility or a more ordered structure. The ratio D1:D2 shifts toward domain 1 and *T*_1_ (^1^H) relaxation times become longer than for the control samples, indicating that relaxation via water is restricted. The three observations can be interpreted as being due to the uncured resin breaking hydrogen bonds between hemicellulose and cellulose and penetrating the interface region of the two domains. In this way, the formation of hydrogen bonds between water and cellulose is restricted, resulting in *T*_1_ (^1^H) relaxation times that are longer compared to those for the unmodified control samples. Since hydrogen bonds are broken down, some of the domain 2 cellulose changes to a domain 1 conformation, similar to the effect of drying in the model presented by Cresswell et al.^47^ A change from cellulose domain 2 to domain 1 could also mean that microfibrils collapse upon each other, which would increase the ratio of the microfibril core to the surface (Table S2).

At the cellulose–hemicellulose interface, the uncured resin has a plasticizing effect on the cell wall, decreasing both *T*_1ρ_ and *T*_1_ (^13^C) relaxation times for cellulose C2, C3, and C5, hemicellulose, and lignin.^54,55^ This plasticizing effect could explain the pliable character of uncured wood.^9,10^ The stark difference between the *T*_1ρ_ relaxation times of C4^D1^ at 89 ppm and C4^D2^ at 84 ppm indicates a phase separation of the two cellulose domains, although it is unlikely that the distance between domains 1 and 2 has increased by more than 30 nm, where spin-coupling would become ineffective.^8^ A more likely explanation is that domain 2 interacts more intensely with the uncured resin than domain 1, e.g., by hydrogen bonding, nonpolar interactions, or as a consequence of steric hindrance.

The separate hemicellulose phase with a much lower *T*_1ρ_ relaxation time seems to be less affected by interactions with the resin. Even after heat curing of Wood A, the *T*_1ρ_ relaxation time of hemicellulose at 21 ppm remains on a small level. The *T*_1_ (^1^H) relaxation time for cured Wood A increases beyond the level of both individual components (i.e., resin and dry wood), indicating the formation of a rigid nanoscale composite at a length scale of >30 nm. This is currently interpreted as a macrofibril that is uniformly penetrated by a cross-linked resin matrix. The range of *T*_1ρ_ relaxation times indicates phase differences within the macrofibril (2–30 nm).

While Wood B shows many similar trends to Wood A, there are also some significant differences (Figure 6). The ratio D1:D2 is less affected for uncured samples and becomes even lower than in the control groups after curing of Wood B. The *T*_1_ (^1^H) relaxation time, which has a value similar to that for Wood A in the uncured state, decreases after curing and reflects a weighted average of *T*_1_ (^1^H) relaxation times of dry wood and cured resin. This is interpreted as a coexistence rather than interaction of both components on a level of >30 nm. On a level of 2–30 nm, cured Wood B might exhibit a more uniform distribution of resin within the cell wall matrix than cured Wood A, since the *T*_1ρ_ relaxation times of Wood B are in a closer range. This suggests that the microfibril and associated hemicelluloses in cured Wood B retain their original structure to a greater extent, with the resin primarily occupying the micropores, whereas in Wood A, the resin has penetrated more extensively into the cellulose–hemicellulose interface, causing greater alterations.

The hemicellulose fraction in cured Wood B seems to be incorporated differently than in cured Wood A, since the *T*_1ρ_ value is twice as long for Wood B. This can be interpreted as being due to the matrix hemicelluloses being more closely incorporated into the resin network in cured Wood B, or as the result of resin agglomerates creating tension in the hemicellulose chain, whereas the hemicellulose signal at 21 ppm is virtually unaffected by heat curing in Wood A. The *T*_1_ (^13^C) relaxation time of lignin constitutes a major difference between Wood A and Wood B. Uncured resin in Wood B exhibits accelerated *T*_1_ (^13^C) relaxation beyond the level that is achieved by water in the control sample. In Wood A, the lignin fraction is much less affected by uncured resin. This may indicate that the resin in Wood B is located closer to the lignin-rich areas in the cell wall, such as the coating of the macrofibril or the middle lamella.

Differences between Wood A and Wood B are evident, but the data presented in this study leave room for interpretation. A criticism of our work would be to state that we have studied only these two samples; analysis of additional samples would allow statistical tests on the significance of the observations, though this is beyond the scope of what we consider to already be a thorough and extensive study. More evidence would be needed to clarify the exact locations of the resin after cell wall diffusion. For example, further solid-state NMR analysis could involve probing ^1^H spin diffusion via ^13^C by applying selective rf pulses. Moreover, further work with ^13^C enriched PUF resin could provide a much greater deal of information by enabling the use of quantifiable (e.g., DP-MAS NMR) and two-dimensional NMR techniques (e.g., CP spin diffusion and refocused INADEQUATE NMR).

## Conclusions

Two sets of resin impregnated wood were dried differently to enable (in Wood A) or restrict (in Wood B) the cell wall diffusion of resin monomers. The resin–wood interactions were monitored on a macroscale (using ASE tests), on a microscale (using SEM), and on a submicroscale (using DSC and NMR).

On a macroscopic level, the BU of Wood A and Wood B differed by approximately 27% and that of *S*_1_ differed by approximately 16% (Table 1). The different behavior of Woods A and B is caused by the different atmospheric conditions in the drying stage, which changes the mechanism of cell wall diffusion for both timbers. Cell wall diffusion occurred in both Wood A and Wood B, as shown by the positive BU (Table 1) and the significant reduction in bound water (Table 2). However, differences on short length-scales are responsible for the different macroscopic swelling behavior.

On a microscopic level, SEM images displayed distinct resin features in the lumen of Woods A and B, indicating differences in the resin’s mobility to diffuse. The micrographs show no visible differences in the number of resin-filled lumens, however, which suggests that the amounts of resin in the cell walls is similar.

The submicroscopic length-scale, analyzed by DSC and NMR, offers an explanation for the observed differences between Woods A and B. However, the NMR results in particular are open to multiple interpretations. The DSC results show that modified wood contains less bound water than unmodified wood. Compared to the unmodified wood, Wood A contains approximately 51% less bound water and Wood B approximately 38% (Table 2). Thus, the resin that is fixed in the cell wall prevents water adsorption to the cellulose and hemicelluloses. This different efficiency in excluding water adsorption explains the different swelling coefficients observed in the ASE tests.

Solid-state NMR observations, in particular ^1^H and ^13^C relaxation time analysis, indicate that the resin in Wood A diffused preferentially to the cellulose–hemicellulose interface, where existing hydrogen bonds are broken down and replaced by a resin matrix. NMR studies in Wood B suggest that the resin diffused preferentially to lignin-rich areas. Hence, in Wood B there might be less resin in direct contact with the microfibril. This explanation would be consistent with the ASE and DSC data, but further research is needed to understand the resin arrangement at the lowest level of scale.

While the influence of cell wall diffusion was evident in this study, future research could provide further insight into the mechanisms involved using variable-temperature NMR studies, experiments with ^13^C enriched PUF resin, and chemical imaging techniques showing the distribution of resin in the cell wall.

## Data Availability

The data sets generated during the current study are available from WRAP, the Warwick Research Archive Portal at https://wrap.warwick.ac.uk/186856/.

## Supporting Information Available

Images of the sample preparation, a representative DSC thermogram, additional SEM images of Wood A and Wood B, drying curves, a ^1^H MAS NMR spectrum of all test groups, representative signal intensity vs contact time curves used to measure relaxation times, additional information on ASE tests, and ^1^H *T*_1ρ_ and *T*_1_ (^13^C) relaxation times in numerical form(PDF)

## Abbreviations

ASE: anti swelling efficiency
BU: bulking coefficient
CP MAS NMR: cross-polarization magic angle spinning nuclear magnetic resonance
DSC: differential scanning calorimetry
HR: heating rate
LU: liquid resin uptake
MC: moisture content
ML: mass loss after soaking in water
SEM: scanning electron microscopy
PUF: phenol urea formaldehyde
Wood A: dried to facilitate cell wall diffusion of resin
Wood B: dried to restrict cell wall diffusion of resin
WPG: weight percentage gain
WR: water ratio
WU: water uptake after soaking in water

## References

[ref1] Churkina G.; Organschi A.; Reyer C. P. O.; Ruff A.; Vinke K.; Liu Z.; Reck B. K.; Graedel T. E.; Schellnhuber H. J. Buildings as a Global Carbon Sink. Nat. Sustain. 2020, 3 (4), 269–276. 10.1038/s41893-019-0462-4.

[ref2] Hill C. A. S. Wood Modification Chemical, Thermal and Other Processes; John Wiley & Sons: London, UK, 2006.

[ref3] Sandberg D.; Kutnar A.; Mantanis G. Wood Modification Technologies - A Review. IForest. 2017, 10, 895–908. 10.3832/ifor2380-010.

[ref4] Zelinka S. L.; Altgen M.; Emmerich L.; Guigo N.; Keplinger T.; Kymäläinen M.; Thybring E. E.; Thygesen L. G. Review of Wood Modification and Wood Functionalization Technologies. Forests 2022, 13 (7), 1004 10.3390/f13071004.

[ref5] Stefanowski B. K.; Spear M. J.; Curling S. F.; Pitman A. J. Properties of Lignia Modified Wood. IOM3 Wood Technology Society Timber 2020, n/a.

[ref6] Jones D.; Sandberg D.; Goli G.; Todaro L. Wood Modification in Europe: A State-of-the-Art about Processes, Products and Applications; Firenze University Press: Florence, IT, 2019.

[ref7] Lande S.; Westin M.; Schneider M. Development of Modified Wood Products Based on Furan Chemistry. Mol. Cryst. Liq. Cryst. 2008, 484, 367–378. 10.1080/15421400801901456.

[ref8] Laborie M. G. Investigation of the Wood-Phenol-Formaldehyde Adhesive Interphase Morphology, PhD Thesis, Virginia Polytechnic Institute and State University, 2002.

[ref9] Shams M. I.; Yano H.; Endou K. Compressive Deformation of Wood Impregnated with Low Molecular Weight Phenol Formaldehyde (PF) Resin I: Effects of Pressing Pressure and Pressure Holding. J. Wood Sci. 2004, 50 (4), 337–342. 10.1007/s10086-003-0570-6.

[ref10] Schwarzkopf M. Densified Wood Impregnated with Phenol Resin for Reduced Set-Recovery. Wood Mater. Sci. Eng. 2021, 16 (1), 35–41. 10.1080/17480272.2020.1729236.

[ref11] Pečnik J. G.; Kutnar A.; Militz H.; Schwarzkopf M.; Schwager H. Fatigue Behavior of Beech and Pine Wood Modi Fi Ed with Low Molecular Weight Phenol-Formaldehyde Resin. Holzforschung 2021, 75 (1), 37–47. 10.1515/hf-2020-0015.

[ref12] Park S.; Venditti R. A.; Jameel H.; Pawlak J. J. Changes in Pore Size Distribution during the Drying of Cellulose Fibers as Measured by Differential Scanning Calorimetry. Carbohydr. Polym. 2006, 66 (1), 97–103. 10.1016/j.carbpol.2006.02.026.

[ref13] Stamm J. A. Dimensional Stabilization of Wood with Carbowaxes. For. Prod. J. 1956, 6 (5), 201–204.

[ref14] Tanaka S.; Seki M.; Miki T.; Shigematsu I.; Kanayama K. Solute Diffusion into Cell Walls in Solution-Impregnated Wood under Conditioning Process I: Effect of Relative Humidity on Solute Diffusivity. J. Wood Sci. 2015, 61 (6), 543–551. 10.1007/s10086-015-1503-x.

[ref15] Tanaka S.; Seki M.; Miki T.; Umemura K.; Kanayama K. Solute Diffusion into Cell Walls in Solution-Impregnated Wood under Conditioning Process III: Effect of Relative Humidity Schedule on Solute Diffusion into Shrinking Cell Walls. J. Wood Sci. 2017, 63 (3), 263–270. 10.1007/s10086-017-1613-8.

[ref16] Tanaka S.; Seki M.; Miki T.; Shigematsu I.; Kanayama K. Solute Diffusion into Cell Walls in Solution-Impregnated Wood under Conditioning Process II: Effect of Solution Concentration on Solute Diffusion. J. Wood Sci. 2016, 62 (2), 146–155. 10.1007/s10086-016-1539-6.

[ref17] Zheng P.; Aoki D.; Seki M.; Miki T.; Tanaka S.; Kanayama K.; Matsushita Y.; Fukushima K. Visualization of Solute Diffusion into Cell Walls in Solution-Impregnated Wood under Varying Relative Humidity Using Time-of-Flight Secondary Ion Mass Spectrometry. Sci. Rep. 2018, 8, 9819 10.1038/s41598-018-28230-2. 29959407 PMC6026143

[ref18] Jakes J. E. Mechanism for Diffusion through Secondary Cell Walls in Lignocellulosic Biomass. J. Phys. Chem. B 2019, 123 (19), 4333–4339. 10.1021/acs.jpcb.9b01430. 31020839

[ref19] Tanaka S.; Seki M.; Miki T.; Umemura K.; Kanayama K. Solute Diffusion into Cell Walls in Solution-Impregnated Wood under Conditioning Process IV: Effect of Temperature on Solute Diffusivity. J. Wood Sci. 2017, 63 (6), 644–651. 10.1007/s10086-017-1658-8.

[ref20] Hill C. A. S.; Forster S. C.; Farahani M. R. M.; Hale M. D. C.; Ormondroyd G. A.; Williams G. R. An Investigation of Cell Wall Micropore Blocking as a Possible Mechanism for the Decay Resistance of Anhydride Modified Wood. Int. Biodeterior. Biodegrad. 2005, 55 (1), 69–76. 10.1016/j.ibiod.2004.07.003.

[ref21] Newman R. H. Solid-State ^13^C NMR Spectroscopy of Multiphase Biomaterials. Polym. Commun. Guildf. 1992, 27 (5), 154–157.

[ref22] Zumbulyadis N. Selective Carbon Excitation and the Detection of Spatial Heterogeneity in Cross-Polarization Magic-Angle-Spinning NMR. J. Magn. Reson. 1983, 53 (3), 486–494. 10.1016/0022-2364(83)90219-6.

[ref23] Tang H. R.; Wang Y. L.; Belton P. S. ^13^C CPMAS Studies of Plant Cell Wall Materials and Model Systems Using Proton Relaxation-Induced Spectral Editing Techniques. Solid State Nucl. Magn. Reson. 2000, 15 (4), 239–248. 10.1016/S0926-2040(99)00064-8. 10772266

[ref24] Newman R. H. Nuclear Magnetic Resonance Study of Spatial Relationships Between Chemical Components in Wood Cell Walls. Holzforschung 1992, 46 (3), 205–210. 10.1515/hfsg.1992.46.3.205.

[ref25] Nishida M.; Tanaka T.; Miki T.; Hayakawa Y.; Kanayama K. Integrated Analysis of Solid-State NMR Spectra and Nuclear Magnetic Relaxation Times for the Phenol Formaldehyde (PF) Resin Impregnation Process into Soft Wood. RSC Adv. 2017, 7 (86), 54532–54541. 10.1039/C7RA11295E.

[ref26] Nishida M.; Tanaka T.; Miki T.; Hayakawa Y.; Kanayama K. Integrated Analysis of Modified Japanese Cypress Using Solid-State NMR Spectra and Nuclear Magnetic Relaxation Times. Cellulose 2019, 26 (6), 3625–3642. 10.1007/s10570-019-02330-2.

[ref27] Kupfernagel C.; Spear M. J.; Pitman A. J.; Ormondroyd G. A. Wood Modification with Phenol Urea Formaldehyde (PUF) Resin: The Influence of Wood Species Selection on the Dimensional Stability. Eur. J. Wood Wood Prod. 2023, 81, 5–19. 10.1007/s00107-022-01893-5.

[ref28] Zelinka S. L.; Lambrecht M. J.; Glass S. V.; Wiedenhoeft A. C.; Yelle D. J. Examination of Water Phase Transitions in Loblolly Pine and Cell Wall Components by Differential Scanning Calorimetry. Thermochim. Acta 2012, 533, 39–45. 10.1016/j.tca.2012.01.015.

[ref29] Metz G.; Wu X. L.; Smith S. O. Ramped-Amplitude Cross Polarization in Magic-Angle-Spinning NMR. J. Magn. Reson. Ser. A 1994, 110 (2), 219–227. 10.1006/jmra.1994.1208.

[ref30] Fung B. M.; Khitrin A. K.; Ermolaev K. An Improved Broadband Decoupling Sequence for Liquid Crystals and Solids. J. Magn. Reson. 2000, 142 (1), 97–101. 10.1006/jmre.1999.1896. 10617439

[ref31] Frye J. S. Comparison of Inversion-Recovery Methods for Measuring Longitudinal Relaxation Rates. Concepts Magn. Reson. 1989, 1 (1), 27–33. 10.1002/cmr.1820010107.

[ref32] Torchia D. A. The Measurement of Proton-Enhanced Carbon-13 T_1_ Values by a Method Which Suppresses Artifacts. J. Magn. Reson. 1978, 30 (3), 613–616. 10.1016/0022-2364(78)90288-3.

[ref33] Furuno T.; Imamura Y.; Kajita H. The Modification of Wood by Treatment with Low Molecular Weight Phenol-Formaldehyde Resin: A Properties Enhancement with Neutralized Phenolic-Resin and Resin Penetration into Wood Cell Walls. Wood Sci. Technol. 2004, 37 (5), 349–361. 10.1007/s00226-003-0176-6.

[ref34] Kamke F. A.; Lee J. N. Adhesive Penetration in Wood - A Review. Wood Fiber Sci. 2007, 39 (2), 205–220.

[ref35] Behr G.; Bollmus S.; Gellerich A.; Militz H. The Influence of Curing Conditions on the Properties of European Beech (*Fagus Sylvatica*) Modified with Melamine Resin Assessed by Light Microscopy and SEM-EDX. Int. Wood Prod. J. 2018, 9 (1), 22–27. 10.1080/20426445.2017.1416738.

[ref36] Donaldson L. A.; Cairns M.; Hill S. J. Comparison of Micropore Distribution in Cell Walls of Softwood and Hardwood Xylem. Plant Physiol. 2018, 178 (3), 1142–1153. 10.1104/pp.18.00883. 30217826 PMC6236611

[ref37] Dieste A.; Krause A.; Mai C.; Sèbe G.; Grelier S.; Militz H. Modification of Fagus Sylvatica L. with 1,3-Dimethylol-4,5-Dihydroxy Ethylene Urea (DMDHEU). Part 2: Pore Size Distribution Determined by Differential Scanning Calorimetry. Holzforschung 2009, 63 (1), 89–93. 10.1515/HF.2009.023.

[ref38] Maloney T. C. Thermoporosimetry of Hard (Silica) and Soft (Cellulosic) Materials by Isothermal Step Melting. J. Therm. Anal. Calorim. 2015, 121 (1), 7–17. 10.1007/s10973-015-4592-2.

[ref39] Zauer M.; Kretzschmar J.; Großmann L.; Pfriem A.; Wagenfuhr A. Analysis of the Pore-Size Distribution and Fiber Saturation Point of Native and Thermally Modified Wood Using Differential Scanning Calorimetry. Wood Sci. Technol. 2014, 48, 177–193. 10.1007/s00226-013-0597-9.

[ref40] Thybring E. E.; Fredriksson M. Wood and Moisture. In Springer Handbook of Wood Science and Technology; Niemz P., Teischinger A., Sandberg D., Eds.; Springer International Publishing: Cham, 2023; pp 355–397.

[ref41] Mai C.; Zhang K. Wood Chemistry. In Springer Handbook of Wood Science and Technology; Niemz P., Teischinger A., Sandberg D., Eds.; Springer International Publishing: Cham, 2023; pp 179–279.

[ref42] Zhao C.; Pizzi A.; Garnier S. Fast Advancement and Hardening Acceleration of Low-Condensation Alkaline PF Resins by Esters and Copolymerized Urea. J. Appl. Polym. Sci. 1999, 74 (2), 359–378. 10.1002/(SICI)1097-4628(19991010)74:2<359::AID-APP18>3.0.CO;2-A.

[ref43] Tomita B.; Hse C. Phenol — Urea — Formaldehyde (PUF) Co-Condensed Wood Adhesives. Int. J. Adhes. Adhes. 1998, 18, 69–79. 10.1016/S0143-7496(97)00047-X.

[ref44] Newman R. H.; Hemmingson J. A. Determination of the Degree of Cellulose Crystallinity in Wood by Carbon-13 Nuclear Magnetic Resonance Spectroscopy. Holzforschung 1990, 44 (5), 351–356. 10.1515/hfsg.1990.44.5.351.

[ref45] Maunu S. L. NMR Studies of Wood and Wood Products. Prog. Nucl. Magn. Reson. Spectrosc. 2002, 40 (2), 151–174. 10.1016/S0079-6565(01)00041-3.

[ref46] Hult E. L.; Larsson P. T.; Iversen T. Comparative CP/MAS ^13^C-NMR Study of Cellulose Structure in Spruce Wood and Kraft Pulp. Cellulose 2000, 7 (1), 35–55. 10.1023/A:1009236932134.

[ref47] Cresswell R.; Dupree R.; Brown S. P.; Pereira C. S.; Skaf M. S.; Sorieul M.; Dupree P.; Hill S. Importance of Water in Maintaining Softwood Secondary Cell Wall Nanostructure. Biomacromolecules 2021, 22 (11), 4669–4680. 10.1021/acs.biomac.1c00937. 34669375 PMC8579401

[ref48] Argyropoulos D. S.; Morin F. G. Probing the Macromolecular Structure of Wood and Pulps with Proton Spin-Lattice Relaxation Time Measurements in the Solid State. Wood Sci. Technol. 1995, 29 (1), 19–30. 10.1007/BF00196929.

[ref49] Schmidt R. G.; Frazier C. E. ^13^C CP/MAS NMR as a Direct Probe of the Wood-Phenol Formaldehyde Adhesive Bondline. Wood Fiber Sci. 1998, 30 (3), 250–258.

[ref50] Terrett O. M.; Lyczakowski J. J.; Yu L.; Iuga D.; Franks W. T.; Brown S. P.; Dupree R.; Dupree P. Molecular Architecture of Softwood Revealed by Solid-State NMR. Nat. Commun. 2019, 10 (1), 4978 10.1038/s41467-019-12979-9. 31673042 PMC6823442

[ref51] Kulasinski K.; Guyer R.; Derome D.; Carmeliet J. Water Adsorption in Wood Microfibril-Hemicellulose System: Role of the Crystalline-Amorphous Interface. Biomacromolecules 2015, 16 (9), 2972–2978. 10.1021/acs.biomac.5b00878. 26313656

[ref52] Terenzi C.; Prakobna K.; Berglund L. A.; Furó I. Nanostructural Effects on Polymer and Water Dynamics in Cellulose Biocomposites: ^2^H and ^13^C NMR Relaxometry. Biomacromolecules 2015, 16 (5), 1506–1515. 10.1021/acs.biomac.5b00330. 25853702

[ref53] Sjöstöm E. Wood Chemistry: Fundamentals and Applications; Academic Press: San Diego, CA, 1994.

[ref54] Bao S.; Daunch W. A.; Sun Y.; Rinaldi P. L.; Marcinko J. J.; Phanopoulos C. Solid State NMR Studies of Polymeric Diphenylmethane Diisocyanate (PMDI) Derived Species in Wood. J. Adhes. 1999, 71 (4), 377–394. 10.1080/00218469908014549.

[ref55] Marcinko J. J.; Devathala S.; Rinaldi P. L.; Bao S. Investigating the Molecular and Bulk Dynamics of PMDI/Wood. For. Prod. J. 1998, 48 (6), 81.

