# Supplementary material for: Investigating Cell Wall Diffusion in Wood Modified with Phenol Urea Formaldehyde Resin in Different Length Scales
Source: Biomacromolecules. 2025 Jan 14;26(2):900–13. doi: 10.1021/acs.biomac.4c01168 (PMC11825066; doi:10.1021/acs.biomac.4c01168)
Supplement: Supplementary file 1 — bm4c01168_si_001.pdf [file bm4c01168_si_001.pdf]

Investigating Cell Wall Diffusion in Wood modified with Phenol Urea Formaldehyde Resin in different Length Scales

Carlo Kupfernagel[1*], Mohammed Rahman[2], Rosalie Cresswell[2], Morwenna J. Spear[3], Andrew Pitman[4], Steven P. Brown[2], Graham A. Ormondroyd[3]

[1]Institut für Holztechnologie Dresden, 01217 Dresden, Germany,

[2]Department of Physics, University of Warwick, Coventry CV4 7AL, UK

[3]BioComposites Centre, Bangor University, Bangor LL57 2UW, UK

[4]BM Trada, Buckinghamshire HP14 4ND, UK

[*]Email: carlo.kupfernagel@ihd-dresden.de

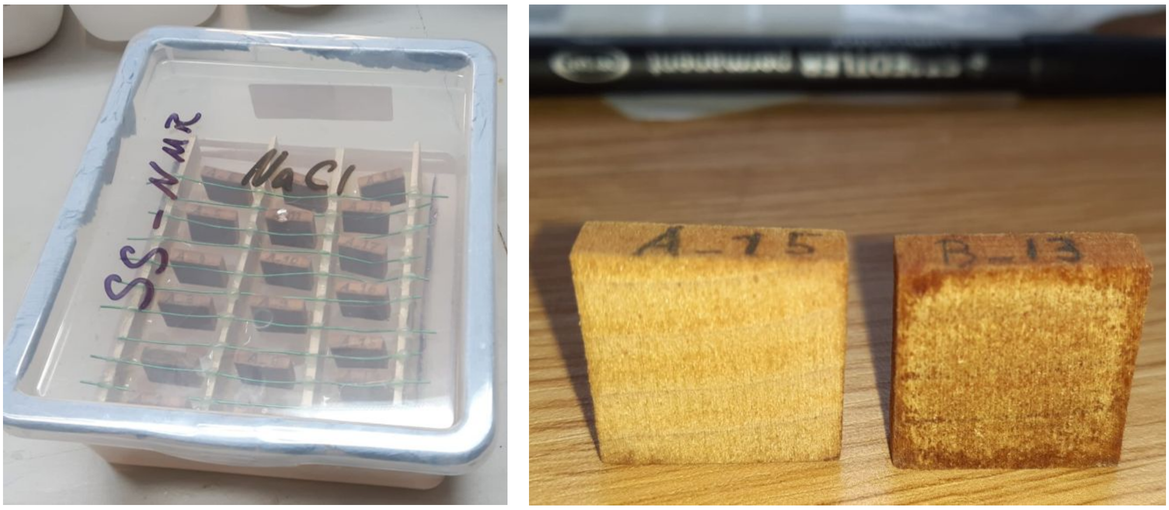

Fig. S1 Wood modification process. (left) drying of resin impregnated samples in a plastic container over a saturated salt solution. (right) Wood A and Wood B after heat curing.

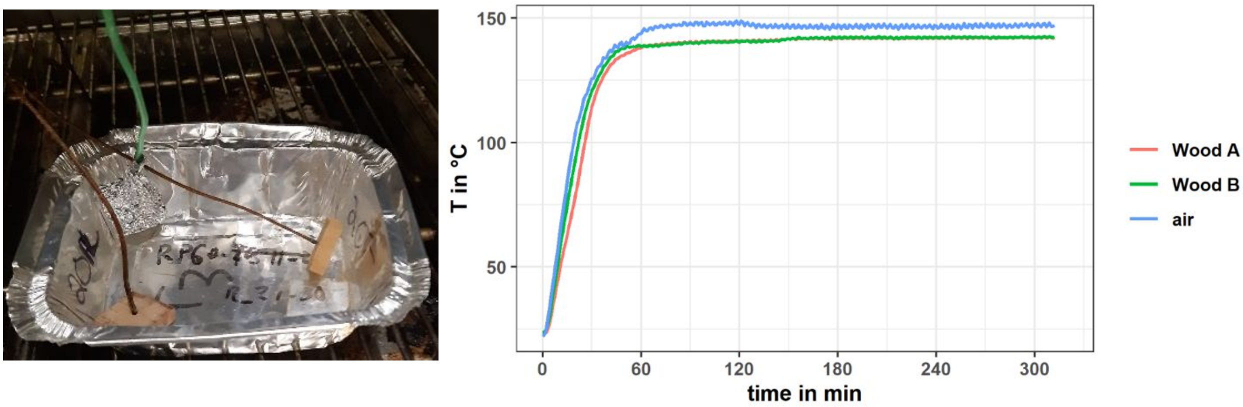

Fig. S2 (left) Thermocouples and wood samples used for the temperature measurement. (right) Temperature profiles during heat curing are similar for Wood A and B, despite slightly different moisture contents prior to cure.

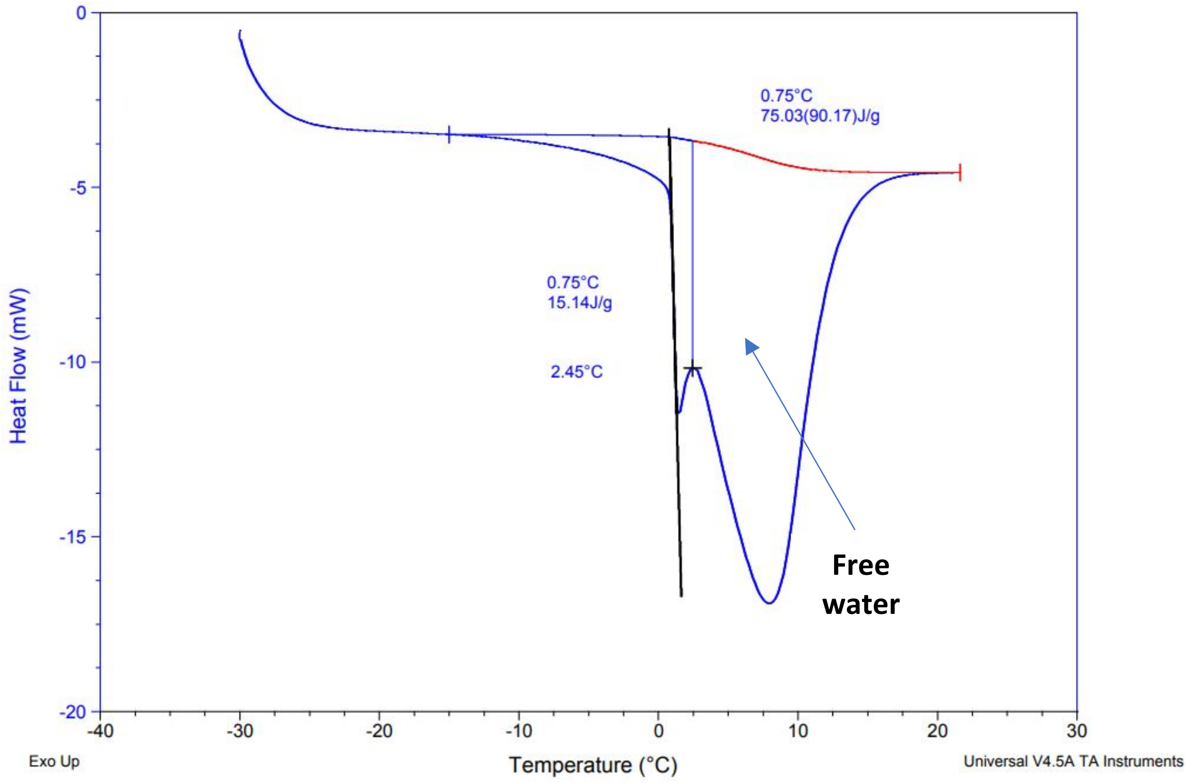

Fig. S3 Example DSC scan of Wood A showing endothermic peaks related to the melting of free water. The heating rate was 10°C/min.

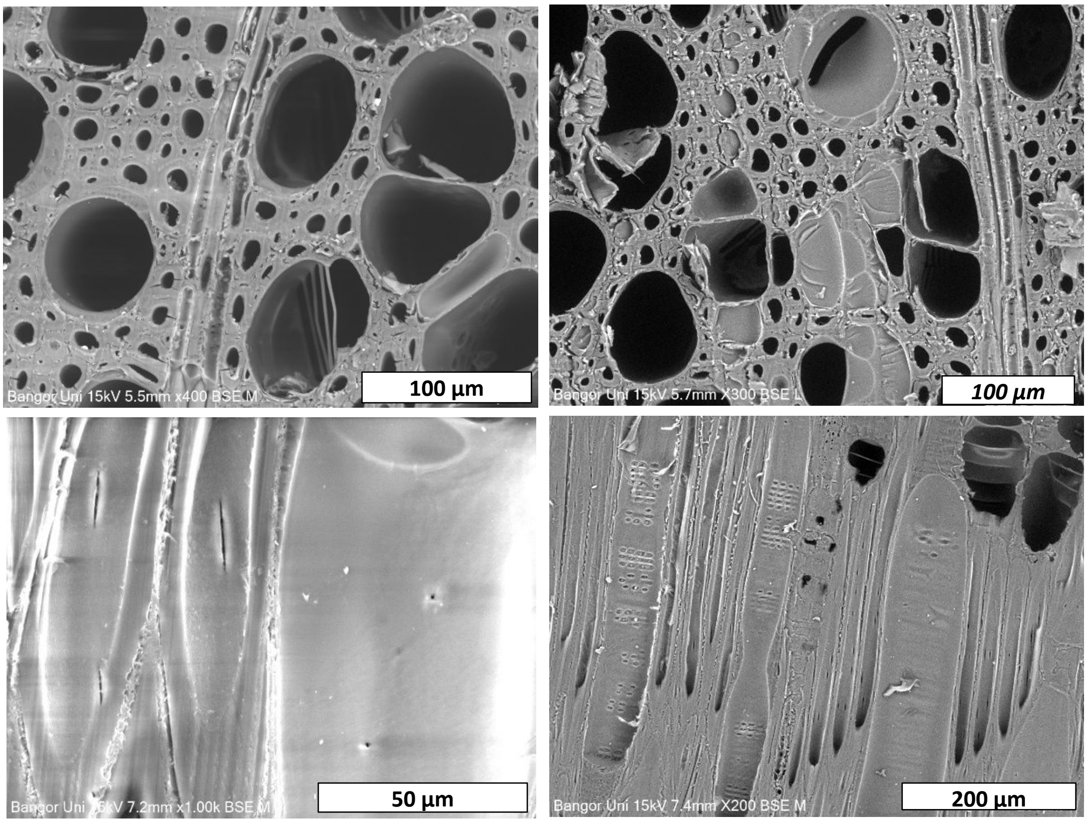

Fig. S4 Scanning electron microscopy images of Wood A in the cured state.

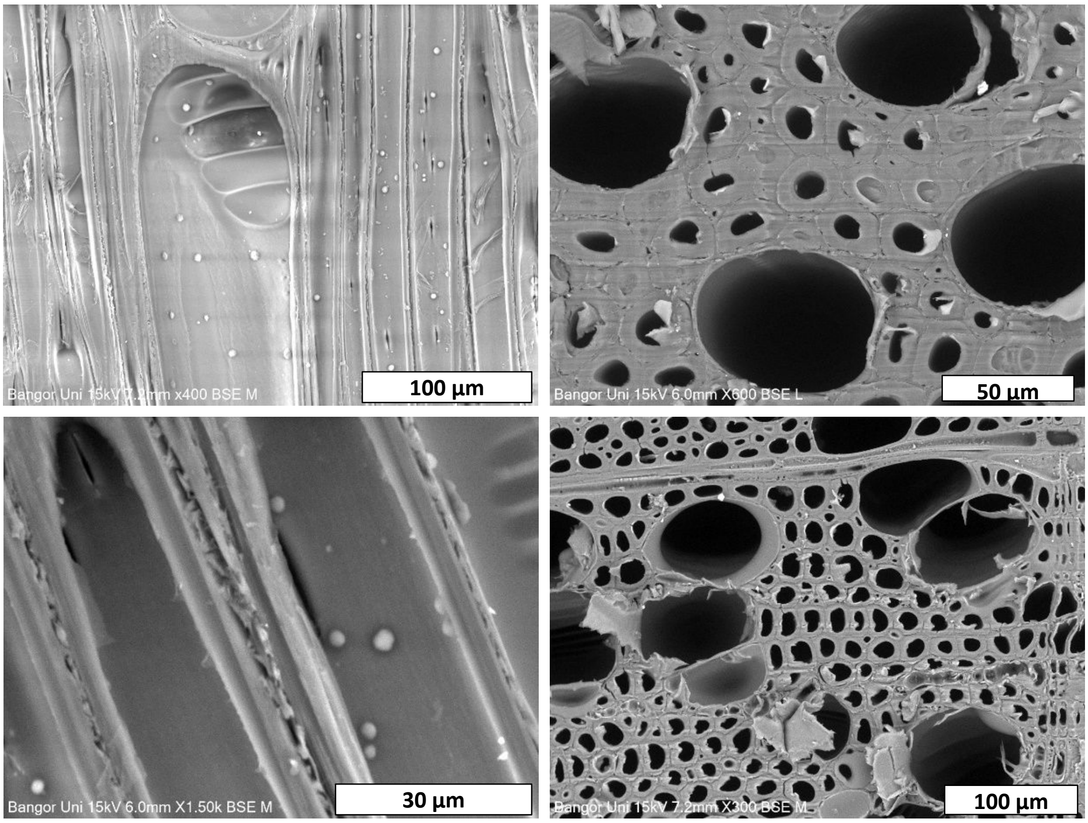

Fig. S5 Scanning electron microscopy images of Wood B in the cured state.

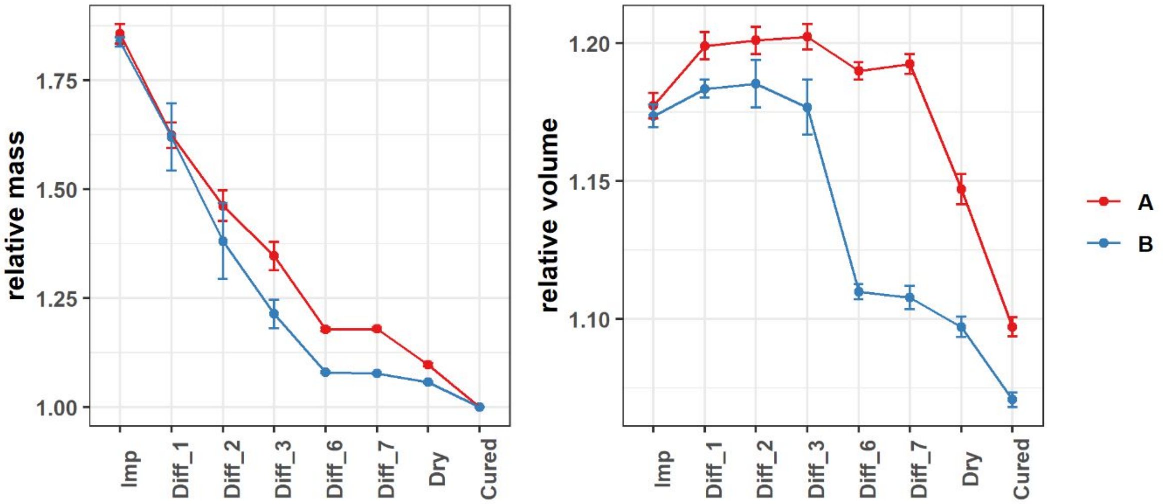

Fig. S6 Relative mass and volume throughout the diffusion and drying stages. Relative mass refers to the cured state and corresponds to the moisture content of a sample. The relative volume refers to the oven dry unmodified state. This better illustrates the initial similarity after impregnation and how Wood A and B separate over the course of the process.

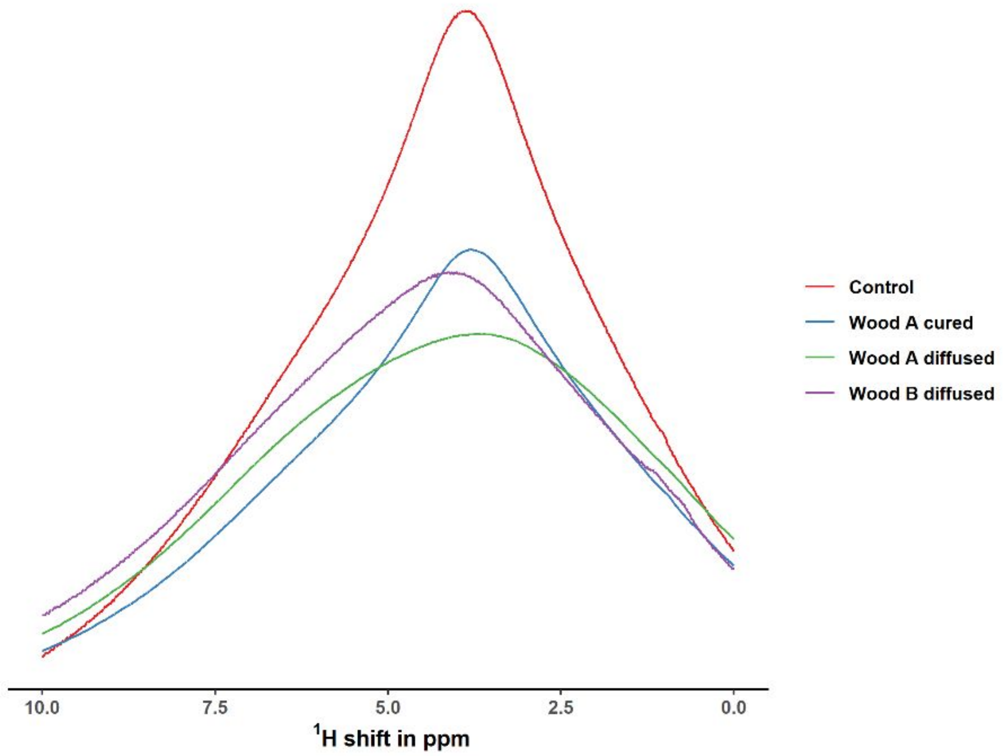

Fig. S7 ^{1}H (500 MHz) MAS (12.5 kHz) NMR spectra of the unmodified control, Wood A and B after diffusion, and Wood A after heat curing.

Equations used to fit the relaxation data in Fig. S8 to S10:

$$I[\tau]=I[0](1-\exp\left(-\frac{\tau}{T_1}\right))$$

$$I[\tau]=I[0](\exp\left(-\frac{\tau}{T_1}\right))$$

$$I[\tau]=I[0](\exp\left(-\frac{\tau}{T_{1\rho}}\right))$$

where $I[\tau]$ is the signal intensity at time τ, $I[0]$ is the initial signal intensity, T_1 is the spin-lattice relaxation time, and $T_1\rho$ is the spin-lattice relaxation time in the rotating frame.

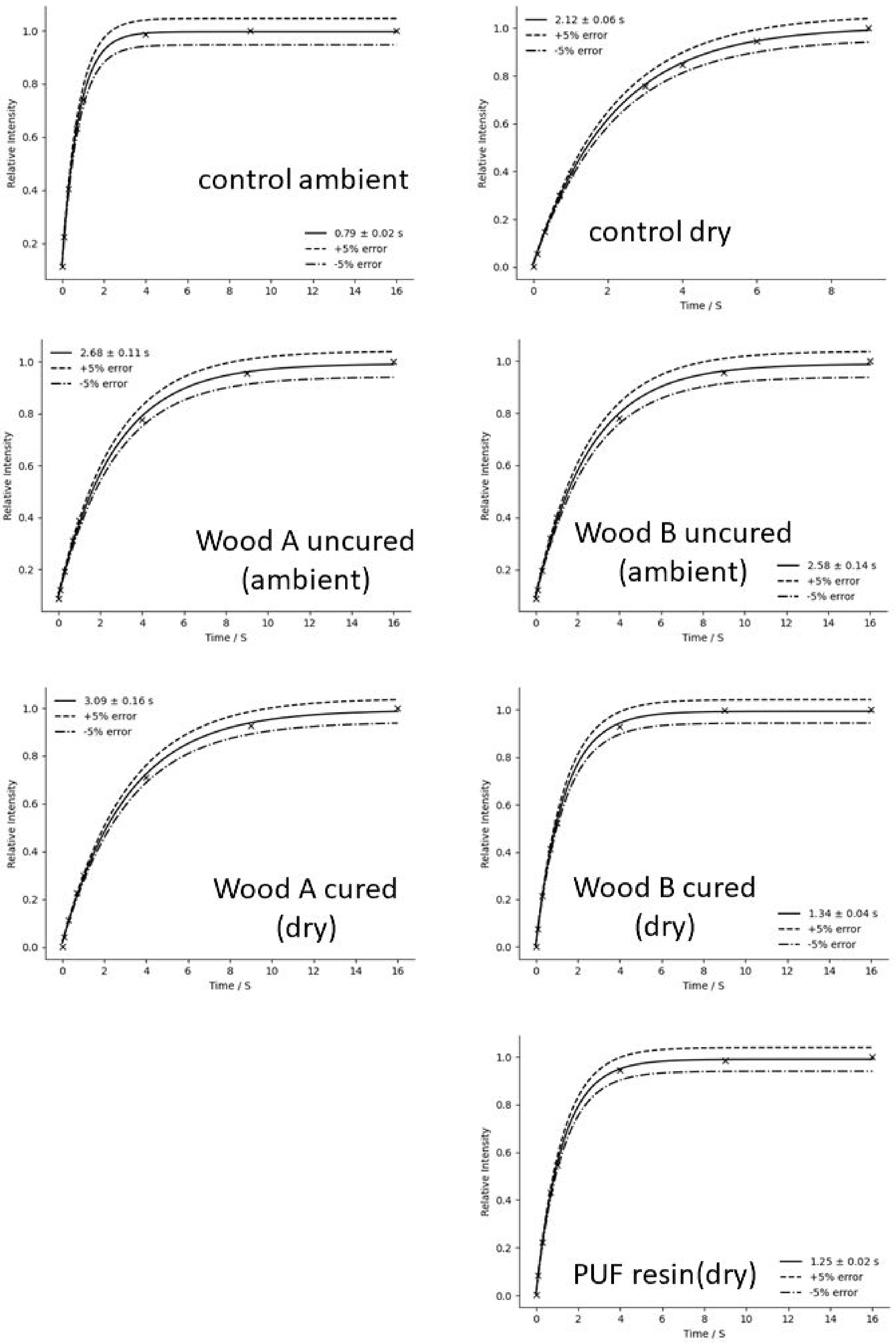

Fig. S8 Plots of signal intensity against time for saturation-recovery experiments used to measure T_1 (^1H) in all samples.

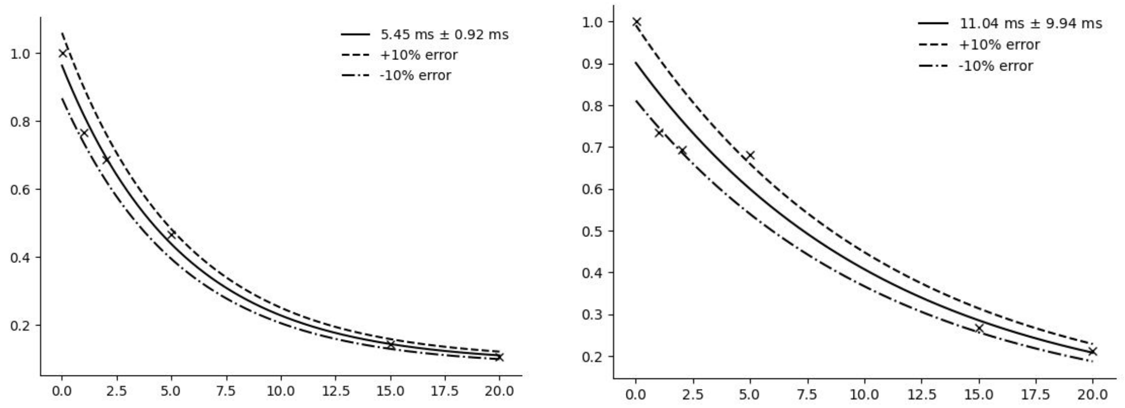

Fig. S9 Example plots of signal intensity against time for ^{1}H-^{13}C CP MAS experiments used to measure $T_1\rho$. The left plot shows an example of a good data fit for the 84 ppm peak in dry unmodified wood. The right plot shows an example of a poor data fit with a high error for the 89 ppm peak in dry unmodified wood.

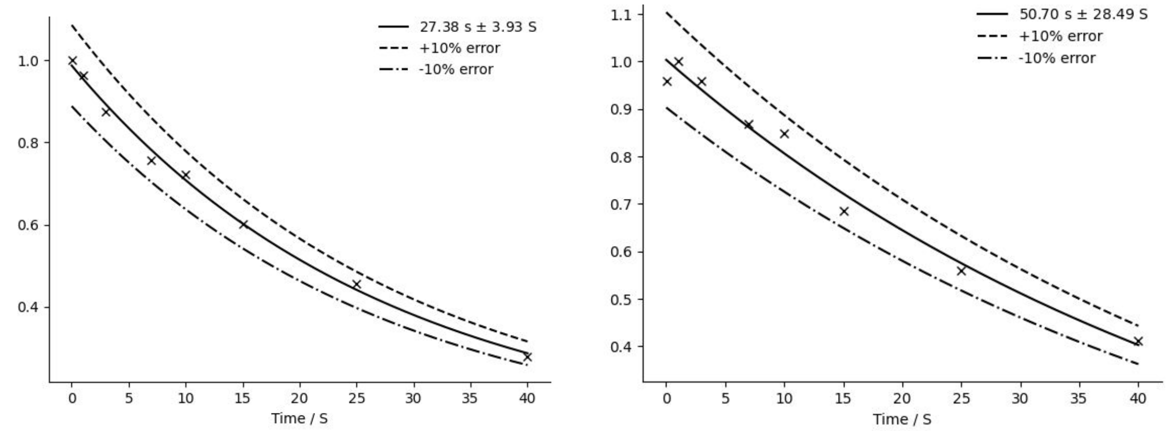

Fig. S10 Example plots of signal intensity against time for T_1 (^{13}C) measurements by the Torchia method. The left plot shows an example of a good data fit for the 84 ppm peak in ambient unmodified wood. The right plot shows an example of a poor data fit for the 89 ppm peak in ambient unmodified wood.

*Table S1 The effect of different diffusion and drying conditions in Wood A and Wood B on their performance in ASE tests.**

	ML1 in %	ML2 in %	ML3 in %	ML total in %	WU1 in %	WU2 in %	WU3 in%
A	−2.65 (0.08)	−1.08 (0.10)	−0.43 (0.08)	−4.11 (0.09)	98.32 (3.08)	102.00 (2.94)	105.14 (3.08)
B	−3.10 (0.17)	−1.09 (0.09)	−0.62 (0.12)	−4.75 (0.19)	97.28 (2.01)	102.17 (2.11)	104.59 (2.45)

** ML1, ML2, ML3 – mass loss due to leaching in water during three cycles of soaking, WU1, WU2, WU3 – gravimetrical water uptake during 3 cycles of soaking.*

Table S2 The effect of different diffusion and drying conditions in Wood A and Wood B on their performance in ASE tests.

Sample	State	MC in %	ratio (D1:D2)
Control (dry)	unmodified	0.00	0.34
Control (ambient)	unmodified	7.48	0.50
PUF	cured	0.00	-
A	cured	0.00	0.41
A	uncured	7.93	0.70
B	cured	0.00	0.26
B	uncured	10.31	0.51

**The ratio D1:D2 is the relative ratio of peak areas in the C4 region of ^{13}C NMR spectrum, where the area under the $C4^{D1}$ peaks at 89 ppm represents, according to the literature, interior cellulose and the area under the $C4^{D2}$ peak at 84 ppm represents, according to the literature, surface cellulose. Historically, this ratio has been referred to as the crystallinity index of cellulose.*

Table S3 Numerical representation of the $T_1\rho$ relaxation times that were measured in this study (see also Figure 4).

$\delta(^{13}C)$ / ppm	sample	state of wood	state of resin	$T_{1\rho}$ in ms	error in ms
21	A	ambient	uncured	2,4	0,8
21	A	dry	cured	2,5	0,9
21	B	ambient	uncured	2,0	0,3
21	B	dry	cured	5,0	1,5
21	Control	ambient	control	0,3	2,9
21	Control	dry	control	7,3	1,3
57	A	ambient	uncured	3,8	0,5
57	A	dry	cured	6,3	1,8
57	B	ambient	uncured	2,7	0,3
57	B	dry	cured	6,3	0,8
57	Control	ambient	control	5,6	0,5
57	Control	dry	control	6,9	0,5
62	Control	ambient	control	5,3	0,2
62	Control	dry	control	5,7	0,6
65	A	ambient	uncured	3,7	0,5
65	A	dry	cured	8,3	0,9
65	B	ambient	uncured	2,6	0,4
65	B	dry	cured	7,1	1,0
65	Control	ambient	control	5,2	0,4
65	Control	dry	control	6,6	0,8
73	A	ambient	uncured	3,7	0,4
73	B	ambient	uncured	2,9	0,4
75	A	ambient	uncured	3,4	0,4
75	A	dry	cured	7,2	1,1
75	B	ambient	uncured	2,6	0,3
75	B	dry	cured	6,4	0,5
75	Control	ambient	control	5,4	0,4
75	Control	dry	control	5,8	0,6
84	A	ambient	uncured	3,4	0,5
84	A	dry	cured	5,5	0,8
84	B	ambient	uncured	2,6	0,2
84	B	dry	cured	6,6	0,6
84	Control	ambient	control	4,9	0,6
84	Control	dry	control	5,5	0,9
89	A	ambient	uncured	4,9	1,0
89	A	dry	cured	9,4	2,9
89	B	ambient	uncured	3,7	0,6
89	B	dry	cured	8,0	1,2
89	Control	ambient	control	4,9	1,1
89	Control	dry	control	11,0	9,9
106	A	ambient	uncured	3,8	0,2
106	A	dry	cured	8,2	0,9
106	B	ambient	uncured	2,9	0,3
106	B	dry	cured	6,9	0,7
106	Control	ambient	control	5,4	0,6
106	Control	dry	control	6,1	0,7
130	A	ambient	uncured	3,3	0,4
130	A	dry	cured	8,3	1,2
130	B	ambient	uncured	2,5	0,3
130	B	dry	cured	6,4	0,4
136	Control	ambient	control	2,4	1,5
136	Control	dry	control	6,2	1,0
153	Control	ambient	control	7,4	2,1
153	Control	dry	control	6,0	1,7
161	A	ambient	uncured	2,9	0,7
161	A	dry	cured	6,1	0,9
161	B	ambient	uncured	1,7	0,2
161	B	dry	cured	6,4	0,2

Table S4 Numerical representation of the T_1 (^{13}C) relaxation times that were measured in this study (see also Figure 5).

$\delta(^{13}C)$ / ppm	sample	state of wood	state of resin	T_1 (^{13}C) in s	error in s
21	A	ambient	uncured	2.9	1.0
21	A	dry	cured	NA	NA
21	B	ambient	uncured	NA	NA
21	B	dry	cured	NA	NA
21	Control	ambient	control	4.0	0.6
21	Control	dry	control	14.1	6.9
57	A	ambient	uncured	2.8	0.4
57	A	dry	cured	NA	NA
57	B	ambient	uncured	0.8	0.3
57	B	dry	cured	1.1	0.2
57	Control	ambient	control	2.2	0.2
57	Control	dry	control	2.6	0.6
62	A	ambient	uncured	3.8	0.8
62	A	dry	cured	3.0	0.3
62	B	ambient	uncured	3.2	0.6
62	B	dry	cured	3.3	0.7
62	Control	ambient	control	2.8	0.6
62	Control	dry	control	3.3	0.9
75	A	ambient	uncured	10.6	2.4
75	A	dry	cured	20.3	7.8
75	B	ambient	uncured	13.1	1.9
75	B	dry	cured	14.9	2.8
75	Control	ambient	control	17.2	0.9
75	Control	dry	control	17.6	1.2
84	A	ambient	uncured	13.8	10.3
84	A	dry	cured	NA	NA
84	B	ambient	uncured	28.3	9.0
84	B	dry	cured	15.5	4.6
84	Control	ambient	control	27.4	3.9
84	Control	dry	control	20.5	2.1
89	A	ambient	uncured	2.2	3.9
89	A	dry	cured	NA	NA
89	B	ambient	uncured	NA	NA
89	B	dry	cured	NA	NA
89	Control	ambient	control	53.9	38.6
89	Control	dry	control	14.9	8.5
